# Marine-Derived Compounds Applied in Cardiovascular Diseases: Submerged Medicinal Industry

**DOI:** 10.3390/md21030193

**Published:** 2023-03-21

**Authors:** Wasim Akram, Mohd Rihan, Sakeel Ahmed, Swamita Arora, Sameer Ahmad, Rahul Vashishth

**Affiliations:** 1 Department of Pharmacology, SPER, Jamia Hamdard, New Delhi 110062, India; 2 Department of Pharmacology and Toxicology, National Institute of Pharmaceutical Education and Research, Mohali 160062, India; 3 Department of Pharmacology, R. V. Northland Institute of Pharmacy, Dadri 203207, India; 4 Department of Food Technology Jamia Hamdard, New Delhi 110062, India; 5 School of BioSciences and Technology-Food Technology, Vellore Institute of Technology, Vellore 632014, India

**Keywords:** marine drugs, cardiovascular diseases (CVDs), atherosclerosis, hypertension, myocardial infarction

## Abstract

Cardiovascular diseases (CVDs) are among the most impactful illnesses globally. Currently, the available therapeutic option has several side effects, including hypotension, bradycardia, arrhythmia, and alteration in different ion concentrations. Recently, bioactive compounds from natural sources, including plants, microorganisms, and marine creatures, have gained a lot of interest. Marine sources serve as reservoirs for new bioactive metabolites with various pharmacological activities. The marine-derived compound such as omega-3 acid ethyl esters, xyloketal B, asperlin, and saringosterol showed promising results in several CVDs. The present review focuses on marine-derived compounds’ cardioprotective potential for hypertension, ischemic heart disease, myocardial infarction, and atherosclerosis. In addition to therapeutic alternatives, the current use of marine-derived components, the future trajectory, and restrictions are also reviewed.

## 1. Introduction

Cardiovascular diseases (CVDs) are one of the most critical conditions affecting human health globally. It includes pulmonary circulation, heart, vascular, and cerebrovascular diseases with higher recurrence and incidence rates [1]. According to 2019 data, CVDs cover 32% with approximately 17.9 million of all deaths around the world. The incidence of CVD-related mortality has grown from 12.1 in 1991 to 18.6 million in 2019 and is expected to increase to 24 million by 2030 [2]. It has been estimated that CVDs will affect approximately 135 million people, with USD 1.1 trillion in costs [3]. Approximately one third of all fatalities worldwide in 2019 were attributable to CVDs, which caused the deaths of 8.9 million women and 9.6 million men. A total of 6.1 million of these fatalities occurred between the ages of 30 and 70. China had the most CVD fatalities, followed by India, the Russian Federation, the United States, and Indonesia [4]. An equal proportion of males and females are affected by CVDs globally, but mortality is higher among women [5]. The goal of the recommendations made by an international group of leaders and professionals in the area is to significantly lower the burden of cardiovascular disease worldwide by 2030 [6]. There are several possible causes of CVDs, such as obesity, smoking, high blood pressure, hyperlipidemia, and diabetes [7]. However, mitochondrial dysfunction is a major factor in CVDs’ pathogenesis via regulating ROS generation [8]. Diseases such as diabetes mellitus, COVID-19 [9], and chronic kidney disease (CKD) [10] also cause CVDs via various pathological pathways in both male and female populations around the world [11]. Currently, several synthetic drugs, including plasminogen activators, angiotensin-converting enzyme blockers, angiotensin-2 receptor blockers, calcium channel blockers, β-blockers, diuretics, and surgery, are used to treat CVDs. However, they are associated with severe adverse effects, including time-dependent effects, hypotension, hypersensitivity, bradycardia, arrhythmia, alteration in several ion levels, e.g., potassium and sodium, and surgical complications [12]. Thus, there is a gap in finding new molecular targets and mechanisms to overcome side effects associated with exitance therapy. Marine drugs have minimal toxicity, no multidrug resistance, and no immune system suppression in CVDs’ patients [13]. Marine natural drugs are more convenient for CVDs due to their high safety profile, numerous biological activities, and natural origin [14]. Furthermore, they demonstrate a wide range of biological actions against various CVDs, including antioxidant, lipid lowering, anti-inflammatory, thrombin inhibition, anti-coagulation, vasodilation, hypoglycemia inducing, antiplatelet activation, and enzyme and ion channel receptor blocking [15]. Thus, marine-derived natural compounds are novel candidates over synthetic drugs for the minimization of adverse effects in CVDs and associated complications. Marine pharmaceuticals are preferable to other products due to their low toxicity, chemical variety, economic effectiveness, and demonstrated therapeutic promise.

## 2. Marine Biodiversity: As Bioactive Reservoirs

The importance of marine natural products (MNPs) in drug discovery, particularly their role in creating current medications, has been well documented [16]. Marine species are the most current source of bioactive natural compounds compared to terrestrial plants and nonmarine microbes. By 2016, 28500 MNPs had been identified, and most had anticancer and cytotoxic properties [17].

The marine environment is a natural habitat for a wide diversity of species with varying physiologies and adaptability to the environment. Out of the more than 33 animal phyla known today, 32 phyla are represented in the marine environment, 15 of which are peculiar to the marine environment [18]. More than 80% of the world’s plant and animal species live in the oceans. Marine organisms include sponges, tunicates, fishes, soft corals, nudibranchs, sea hares, opisthobranch, mollusks, echinoderms, bryozoans, prawns, shells, sea slugs, and marine microorganisms are sources of bioactive compounds [19,20]. Marine ecosystems provide a rich reservoir of novel bioactive chemical entities with significant medicinal potential [21]. The diversity of such molecules is distinct, and their development is encouraged by the chemical and physical circumstances of the sea. It is well known that marine species can create bioactive chemicals to protect themselves against unusual environmental circumstances such as high salt, reactive oxygen species, photodynamic damage, and high temperatures [22]. The ancient Greek, Byzantium, and Mediterranean cultures used marine animals for therapeutic reasons. Since marine invertebrates have become so significant in medical practice, much work has been committed to utilizing them. The medicinal or therapeutic properties and how raw materials are handled and delivered were recorded in ancient literature. Several marine invertebrates have been utilized as fresh or dried meat in beverages, liquids, crushed goods, soups, and ointments [23]. Traditional Chinese medicine has also contributed to marine drug research. Local diaries, folk recipes, bibliographies, early prescriptions, and dietary advice have contributed to our comprehensive grasp of marine medicines and other species. All of this information, as well as subsequent discoveries, may be found in the Chinese marine *Materia medica* [24]. Despite over 250 years of marine research, over 91% of marine creatures still require a complete description. Since the earliest marine animals appeared around 3500 million years ago, adverse conditions fostered the evolution of a diverse range of bioactive chemicals to resist environmental stress [25].

The birth of marine drugs occurred in the 19th century, once biotechnology arose as a science that offered direction to the research of marine medication creation. These marine-derived medications have been utilized to treat various ailments, including cancer, heart disease, diabetes, and neurodegenerative disease [26]. Over the last five decades, evidence has accumulated that marine-based plants and microbes have a greater potential for cancer treatment. For example, cytarabine, eribulin mesylate, brentuximab vedotin, and trabectidine are marine-based drugs used to treat leukemia, metastatic breast cancer, soft tissue sarcoma, and ovarian cancer [21]. Currently, ≥26,680 have been isolated and identified from marine-derived sources, with an average addition of 1000 per year. Due to the specificity of bioactive compounds toward the targets, they are beneficial in cancer therapy targeting cancerous cells [27].

### Classification of Marine Drugs

**Figure 1:**
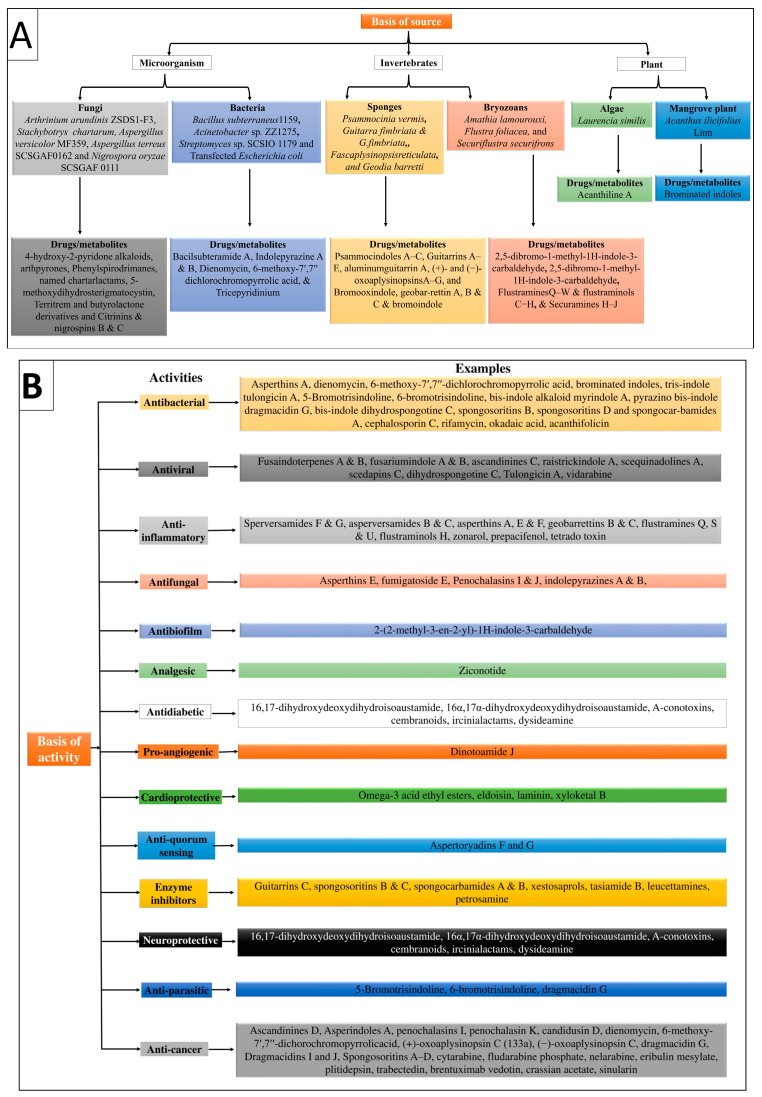
Illustration of the classification of marine-derived compounds based on (**A**) sources and (**B**) activity.

Marine drugs are classified based on their sources and pharmacological actions, compiled in Table 1, Figure 1A,B.

## 3. Role of Marine Drugs in CVDs Management

### 3.1. Hypertension

Hypertension is one of the most severe problems among all CVDs, and is responsible for stroke, ischemic heart disease, dementia, chronic kidney disease, and other CVDs [62]. According to 2019 age-standardized prevalence data, 32% of women and 34% of men aged 30–79 worldwide had hypertension [63]. Many marine natural compounds, including bioactive molecules, chito-oligosaccharide derivatives (COS), and phlorotannins, were obtained from marine species and are potential leads for ACE inhibitors and evolved as nutraceutical medicinal compounds for the treatment of hypertension [64,65]. Natural marine ACE inhibitors are being studied as alternatives to synthetic drugs to avoid several serious side effects and hold a significant potential to become new therapeutic options for the treatment of hypertension [66]. Biopeptides or ACE-inhibitory peptides derived from fish proteins are often made under controlled circumstances by proteolyzing marine proteins advanced for the treatment of hypertension [67]. Furthermore, marine red algae *Gracilariopsis lemaneiformis* have been identified as producing several marine-based new ACE inhibiting peptides, FQIN [M(O)] CILR and TGAPCR, discovered by LC-MS/MS screening in *G. lemaneiformis* protein hydrolysates. These peptides significantly decreased systolic and diastolic blood pressure (DBP) in the spontaneously hypertensive rat model [68]. In the same direction, Sato M. et al. identified seven peptides: Val-Tyr, Ile-Tyr, Ala-Trp, Phe-Tyr, Val-Trp, Ile-Trp, and Leu-Trp from hydrolysates of wakame (*Undaria pinnatifida*) brown seaweed using three steps, HPLC and liquid chromatography-mass spectroscopy. Four of seven seaweed-derived peptides (Val-Tyr, Ile-Tyr, Phe-Tyr, and Ile-Trp) significantly reduced systolic blood pressure in spontaneously hypertensive rats at a dose of 1 mg/kg. This offers a possible source of new AEC inhibitors as antihypertensives [69]. In addition, Sun et al. also identified two Phe-Gly-Met-Pro-Leu-Asp-Arg (FGMPLDR; MW 834.41 Da) and Met-Glu-Leu-Val-Leu-Arg (MELVLR; MW 759.43 Da) ACE inhibitory peptides from the protein hydrolysate marine macroalga of *Ulva intestinalis.* In silico and in vitro molecular docking studies revealed these two peptides have ACE binding and inhibitory activity [70].

**Figure 2:**
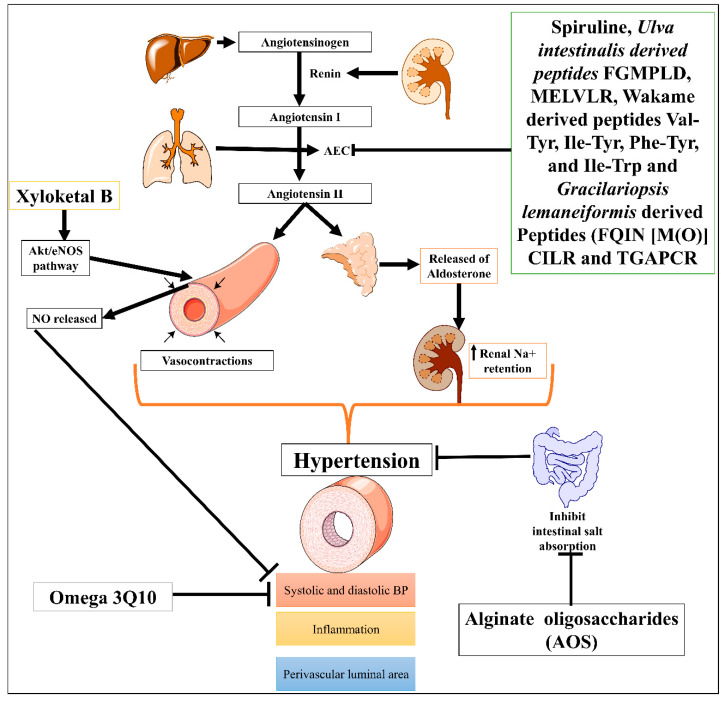
Possible mechanism of different marine-derived compounds in CVDs.

One of the most well-known marine-derived compounds is alginate oligosaccharides (AOS) that offer protection against perivascular inflammation, reduction in the vascular luminal area, and hemodynamic alterations of pulmonary hypertension in the rat produced by monocrotaline (MCT) model via downregulating P-selectin [71]. Another study demonstrated that omega-3 Q10, a polyunsaturated fatty acid (n3-PUFA) formulation, appears to be more effective than soybean oil supplementation at reducing diastolic blood pressure and associated symptoms with hypertension in older adults [72]. Moreover, mangrove fungus-isolated xyloketal B showed phenylephrine (Phe)-induced contractions induced hypertension protection by decreasing the systolic and diastolic blood pressure via enhancing endothelial NO release through the Akt/eNOS pathway [51]. In addition, a controlled trial study conducted by Sámano MJ et al. evaluated the combination of *Spirulina (Arthrospira) maxima* (filamentous, gram-negative cyanobacterium) with angiotensin-converting enzyme (ACE) inhibitors in patients with systemic arterial hypertension (SAH) and accessed its effects on endothelial damage and oxidative stress. Results showed that *Spirulina* significantly reduced systolic blood pressure, increased anti-oxidant level (glutathione peroxidase activity and oxidized glutathione), and decreased endothelial damage markers (sVCAM-1, sE-selectin, and endothelin-1) [73]. It has other properties such as antiviral, anti-dyslipidemic, and antioxidant [74]. Low molecular mass potassium alginate (L-PA), brown algae, shows an antihypertensive effect on DOCA salt-induced hypertension in rats (Figure 2) [75]. Overall, data suggested that marine-derived compounds have the potential to cure hypertension, but a detailed mechanistic study is still needed. Moreover, Therapeutic potential of marine drugs in CVDs management has been tubulated in Table 2.

### 3.2. Atherosclerosis

Atherosclerosis is a chronic, inflammatory, progressive cardiovascular disease that results from ongoing blood vessel damage brought on by hyperlipidemia and increased cholesterol levels [93]. Marine-based derived compounds have been effective against atherosclerosis since ancient times. These compounds have advantages over synthetic compounds in atherosclerosis due to greater effectiveness and lower side effects [94]. Marine-derived algal polysaccharides are the active ingredients in products made from marine sources that have a hypolipidemic impact and cure atherosclerosis.

**Figure 3:**
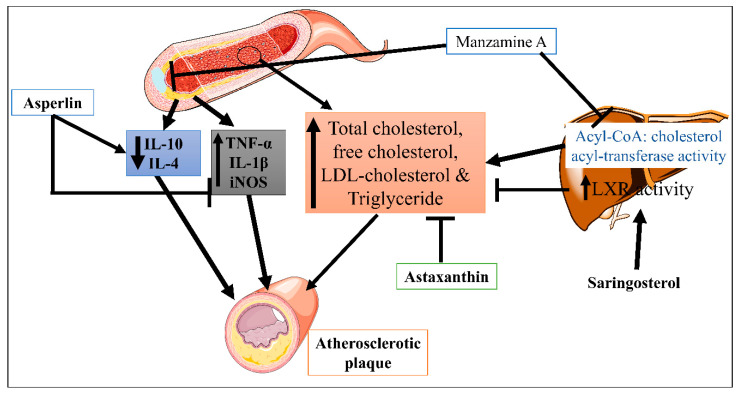
Mechanisms of Manzamine A, Astaxanthin, and Asperlin in CVDs.

Saringosterol, a phytosterol derived from the edible marine seaweed *Sargassum fusiforme*, has high and selective liver X receptor (LXR) activity [95]. Yan et. al. reported that saringosterol treatment reduced the burden of atherosclerotic plaques while having no negative effects on the liver of apoE-deficient rats. Saringosterol reduces cholesterol homeostasis disruption, influencing atherosclerosis’s progression [79]. However, asperlin is derived from the marine fungus *Aspergillus versicolor* LZD4403 and possesses antifungal and anti-inflammatory properties. Zhou Y et. al. reported that asperlin has atheroprotective potential in vitro and in vivo. Results indicated that asperlin treatment significantly reduced inflammatory cytokines (iNOS, IL-1β, and TNF-α), increased protective cytokines (IL-10 and IL-4), and reduced aortic dilation and atherosclerosis plaque formation in the aorta [77]. This suggested that the anti-inflammatory properties of asperlin could be beneficial against atherosclerosis. Manzamine A is a naturally occurring alkaloid obtained from the sea sponge *Acanthostrongylophora ingens* [96]. In atherosclerosis, Eguchi et al. conducted a study where Manzamine A suppressed acyl-CoA: cholesterol acyl-transferase activity in hamster ovary cells. In addition, Manzamine A treatment significantly reduced the serum level of total cholesterol, free cholesterol, LDL-cholesterol, triglyceride, and atherosclerotic lesion formation in apolipoprotein E (apoE)-deficient mice [80]. Astaxanthin is a xanthophyll pigment obtained from microalgae, fungi, complex plants, seafood, and flamingos. As an antioxidant with anti-inflammatory characteristics, it has the potential to be used as a treatment for atherosclerotic cardiovascular disease [97]. Yang Y et. al. demonstrated the hypocholesterolemic effect of astaxanthin via reducing total plasma cholesterol, TG and increased LDL receptor (LDLR), 3-hydroxy-3-methylglutaryl CoA reductase, and sterol regulatory element binding-protein 2 (SREBP-2) and greater mature SREBP-2 protein apoE(-/-) mice (Figure 3) [81]. In high-fat diet mice, Xyloketal B also protects against atherosclerosis through a strong antioxidant effect [78].

Moreover, there are several major causes of atherosclerosis. However, thermo-inflmation plays a crucial role in atherosclerosis pathogenesis via influencing the plague formation. Thrombo-inflammation refers to the complex cascading interaction between the blood coagulation process and inflammation in the pathogenesis of CVDs [98]. The formation of arterial thrombosis is mostly caused by platelet adhesion under high shear stress, which arises in stenotic atherosclerotic arteries [99]. Meanwhile, platelet-activating factor (PAF) is a powerful lipid mediator that acts through PAF/PAF-R pathways and is a key player in inflammation by recruiting neutrophils and activating platelets in the development of atherosclerosis [100].

Several marine-derived drugs have been investigated to inhibit thrombo-inflammation in CVDs. Fascaplysin is a Fijian marine sponge derived from the genus *Fascaplysinopsis* [101], which is a kinase inhibitor with anti-thrombotic properties via inhibiting GPIIb/IIIa activation, platelet aggregation, and thrombus formation [83]. Another cyclodepsipeptide marine compound Isaridin E derived from the *Amphichorda feline* (*Beauveria feline*) fungus [102], demonstrated the dose-dependent inhibition of platelet activation, aggregation, and secretion. However, it does not have any effect against thrombin- or collagen-induced platelet aggregation. Isaridin E also showed an antithrombotic effect without increasing bleeding time in a dose-dependent manner against the FeCl_3_-induced carotid mouse model [84]. F-fucoidan (FD) is a polysaccharide compound derived from the brown alga *Laminaria japonica* that also shows an antithrombotic effect through shortening the blood lysis time, H2O2 expression stimulation, and H2O2 released after induction of PGI2 production and might be effective in CVDs’ patients [103]. The anti-thrombotic and anti-atherosclerotic properties of marine-derived omega 3 polyunsaturated fatty acids (n-3 PUFA) may help to reduce heart failure by lowering the risk of ischemic heart disease. It is known that n-3 PUFA enhances plasminogen activator inhibitor-1 by lowering fibrinogen and decreasing platelet-derived thromboxane A2 (TXA2), which increases platelet aggregation and vasoconstriction [104]. Therefore, So, overall, it seems like marine-based drugs could be used to treat atherosclerosis, but a more detailed mechanistic study is still needed.

### 3.3. Myocardial Infarction (MI)

MI occurs due to the occlusion of the coronary artery, leads to a shortage in oxygen and nutrients, and causes irreversible necrosis and death of cardiomyocytes [105]. It is the major cause of death and disability among other CVDs worldwide [106]. Using marine-derived metal nanoparticles, a novel method for treating thrombus dissolution and myocyte healing in infarcted areas (myocardial infarction) [107]. The anti-myocardial infarction activity of the gold nanoparticles (GNPs) was an innovative method in which cyanobacterial extract, GNP solution, and a combination of both were developed [85]. Omega-3 polyunsaturated fatty acids (PUFA), a marine compound, have shown beneficial benefits on myocardial infarction by reducing MI size in experimental and clinical research (Figure 3) [104]. Docosahexaenoic acid (DHA) is a long-chain omega-3 PUFA obtained from the marine source that has shown a protective effect against myocardial infarction [87]. An in vivo study of DHA in a rat model showed a protective effect against MI at 5 g/kg [108]. There are few marine-derived compounds in MI that have been investigated until now. Thus, in addition, a more detailed mechanistic study is needed.

### 3.4. Ischemic Heart Disease (IHD)

IHD is an inadequate blood supply of the coronary artery to the myocardium. Endothelial dysfunction is the main involvement in the mechanism of IHD [109]. It is the main cause of morbidity and mortality among all CVDs globally [110]. A 2016 report states it is responsible for 9 million deaths worldwide [111]. Marine-derived drugs are better than synthetic drugs to treat IHD due to their affective action and better results [104]. Histochrome, a sodium salt of echinochrome A, is a marine drug found as a common sea urchin pigment. It is a powerful and biosafe cell-priming agent that prevents cardiac progenitor cells (CPCs) from cellular apoptosis via the downregulation of BCL2-associated X (Bax) cleaved caspase-3, and phosphorylated histone, whereas upregulation of Bcl-xL and B-cell lymphoma 2 (Bcl-2) proteins, utilizing patient-derived human CPCs in treating heart disease [112]. In vitro study of echinochrome A (Ech A), a naturally occurring pigment from sea urchins, showed marine anti-thrombotics, especially sulfated polysaccharides, are relevant due to their distinct modes of action and absence of bleeding. Their distinct modes of action as an antithrombotic are due to the high negative charge that sulfation imparts, which enables them to interact with proteins and molecules involved in vital biological processes such as coagulation [113]. In addition, both polysaccharides Enteromorpha prolifera polysaccharides (EPPs), produced from green algae, and fucoidan, extracted from brown algae, have anti-oxidant, lipid-lowering, and antiangiogenic properties [114]. Alginate (ALG), mostly derived from brown seaweed, can lower TC, TG, and LDL-C serum levels and upregulate HDL-C concentrations, making it an effective treatment for coronary artery disease [15].

### 3.5. Cardiac Stroke

Cardiac stroke is the most severe complication of CVDs, causing sudden death. CVDs are mostly caused by cardiac arrest or stroke in individuals with elevated blood pressure, high cholesterol, obesity, increased blood glucose levels, and weight gain [115]. Natural compounds derived from marine sources have already been regarded as lead molecules for treating CVDs and cardiac arrest due to their varied chemical compositions and pharmacological characteristics [116]. A carotenoid molecule called fucoxanthin, obtained from brown algae, prevents lipids’ oxidation and buildup [117]. Fucoxanthin protects against cardiac stroke by regulating metabolic syndrome [118]. Another carotenoid, astaxanthin, showed a positive effect in cardiac stroke via the modulating number of biological processes, including the reduction in inflammation, augmentation of oxidative stress, enhancement of antioxidants, and the modification of lipid and glucose concentrations via suppressing TLR4/NF-κB/ROS signaling pathway [119]. A new type of unique structure called Xyloketal B contains a marine component derived from *Xylaria* species. Xyloketal B can benefit cardiac stroke due to its protective effect in the two-clip stroke-prone hypertensive model [120].

### 3.6. Cardiac Arrhythmia

Cardiac arrhythmias account for 10%–15% of fatalities, making them a substantial reason for morbidity and mortality worldwide [121]. Tetrodotoxin (TTX) is a marine compound obtained from the actinomycetes of marine sediments and has a beneficial effect on cardiac arrhythmia. It is also known as the puffer fish toxin that prevents sodium channels in excitable neurons [122]. It has also shown an antiarrhythmic effect in combinatorial therapy with lidocaine [123].

Many toxins, including tetrodotoxin, saxitoxin, brevetoxins, antillatoxin, conotoxins, and cnidarians, are found in marine species such as pufferfish, shellfish, sea anemones, and cone snails, are voltage-gated sodium channels (VGSCs) blockers, and show protective effects against cardiac arrhythmia [124]. Other marine drugs, omega-3 fatty acids eicosapentaenoic acid and docosahexaenoic acid, have shown antiarrhythmic effects against various arrhythmic disturbances, including atrial fibrillation and ventricular arrhythmia [125]. Eicosapentaenoic acid shows antiarrhythmic activity when added to the superfusate before adding the toxins, including ouabain, lysophosphatidylcholine, high Ca^2+,^ acylcarnitine, β-adrenergic agonist, and the Ca^2+^ ionophore [90]. Botulinum toxin is obtained from the marine source *Clostridium botulinum*. *Clostridium botulinum* is a Gram-positive anaerobic spore-forming bacterium found in marine environments [126]. The botulinum toxin (BoNT/A1)–chitosan nanoparticles (BTN) formulation inhibits arrhythmia caused by sodium, calcium, and potassium channel activation [89].

### 3.7. Cardiac Dysfunction

Chronic cardiac dysfunction is caused by contractility overload on the heart myocardium. Different etiologies may favor existing compensatory mechanisms such as excentric (dilatation) and concentric hypertrophy. Chronic left ventricular dysfunction is the most prevalent complication of MI. Chronic cardiac dysfunction worsens left ventricular ejection fraction and stroke volume as dilatation progresses, eventually leading to heart failure [127]. Retinoid receptors play a crucial role in several diseases, including diabetes [128], cancer [129], and CVDs [130]. A research study reported that the retinoid receptor is essential for heart function. Moreover, tamoxifen-induced myocardial specific RARα deletion (RARαKO) mice showed significant diastolic dysfunction, increased intracellular ROS, NOX2 (NADPH oxidase 2), NOX4 and decreased antioxidant level (SOD1 and SOD2). This effect is reversed by overexpression of retinoid receptors [131]. In addition, Guleria RS et al. also demonstrated that retinoid receptors play a role in diabetic-induced cardiomyopathy [132]. In the same way, zeaxanthin heneicosylate (ZH) extracted from microalgae *Dunaliella salina* significantly reduced plasma biochemical alteration (AST, ALT, urea, and creatinine level), pro-inflammatory level (IL-6, NF-κB, and iNOS), antioxidant level (SOD), and histological changes in D-galactose-induced cardiac dysfunction rats through stimulating the retinoid receptors [92]. There are only a few studies on cardiac dysfunction; thus, detailed mechanistic studies are needed.

### 3.8. Heart Valve Disease or Valvular Heart Disease

Valvular heart disease (VHD) is a cluster of frequent cardiovascular disorders that account for 10–20% of all cardiac surgical operations in the United States. Heart valve problems include regurgitation (valve flaps do not close properly), stenosis (narrowed valve opening), and atresia (valve does not have a proper opening). Fucoxanthin is a marine carotenoid obtained from the seaweed microalgae *Phaeodactylum tricornutum* and possesses antioxidant and anti-inflammatory properties [133]. A report by Chiang et al. demonstrated the protective potential of heart valves in heart valve interstitial cells and dogs. Results showed that fucoxanthin treatment significantly reduced H_2_O_2_-induced ROS level, DNA damage, cell survival, and protein-related apoptosis and calcification expression via modulating the Akt/ERK pathway. In addition, long-term (0.5 to 2 years) supplementation to the dog also improved the left atrium to aortic (LA/AO) dimension ratio and E/e value (indicate mitral valve inflow, mitral valve leakage, and left ventricular diastolic dysfunction) [91]. This suggests that marine-derived compounds hold a diverse therapeutic potential. In addition, marine drugs which hold biological effects in CVDs tubulated in Table 3. 

## 4. Clinical Trial Studies of Marine-Derived Drugs in CVDs

Several marine-derived drugs, such as astaxanthin, alginate, eicosapentaenoic acid, etc., have been approved for clinical trial studies in various CVDs. These are effective when used with CVDs patients in clinical trial studies. However, more studies are required to collect data to prove that marine drugs provide a better therapy for CVDs. All the clinical studies on marine-derived drugs for CVDs are compiled in Table 4.

## 5. Marine Lipid Bioactive Compounds with Potent Cardio-Protective Properties

Omega-3 polyunsaturated fatty acids (PUFA), carotenoids, lipid vitamins, and polar lipid bioactives from fish sources have been shown to have potent biological effects against inflammation and CVDs [157]. Fish is a good source of bio-functional marine polar lipids (PL), rich in n-3 PUFA, and have powerful antithrombotic, anti-inflammatory, and cardio-protective properties. The amphiphilic features of bioactive fish PL, such as several phospholipids and glycolipids bearing n-3 PUFA in their structure, resulted in a significant increase in the bioavailability of n-3 PUFA content [157]. It has been demonstrated that n-3 PUFA-rich fish lowers the incidence of inflammation-related CVDs. Additionally, it has anti-inflammatory and anti-thrombotic characteristics and transports n-3 PUFA to different bodily organs more effectively than triglycerides [158]. In addition to n-3 PUFA, other fish-lipid bioactive nutrients such as fish carotenoids, lipid vitamins A, D, and E, and polar fish lipids (glycolipids and phospholipids) have also shown anti-thrombotic, anti-inflammatory, and antioxidant, cardioprotective effects and reduced CVD risk [158,159]. Polar lipid fractions of sea bass (*Dicentrarchus labrax*) and gilthead sea bream (*Sparus aurata*) treated with fish feed enhanced with olive pomace showed powerful antithrombotic properties [160]. The cold-water marine fish cod (*Gadus morhua*) is a major source of fat-soluble vitamins, EPA and DHA, and high-quality protein. Cod is a lean fish that maintains its fat reserves in the liver as triacylglycerols. It has been reported to possess cardioprotective effects against atherosclerosis and platelet aggregation [161]. Sardines (*Sardina pilchardus*) are a significant Mediterranean commercial fish that reserves fats in the tissue as triacylglycerols. Sardines have shown a potent cardioprotective effect against platelet aggregation or PAF-induced platelet aggregation [162]. Microalgae are abundant sources of bioactive lipids, including polar lipids and omega-3 and -6 PUFA, which have strong anti-inflammatory properties in CVDs [163].

It has been shown that dietary polar lipids of marine origin can either directly inhibit PAF-binding on a particular cell membrane receptor for PAF or indirectly influence the phospholipid microenvironment in those membranes. These lipids could also prevent PAF production, bringing blood PAF levels to homeostatic levels, which has several anti-inflammatory and anti-atherogenic health effects for cardiovascular [159]. *Spirulina*-extracted lipid substances, including lipid extracts, phycocyanin protein, phycocyanobilin (PCB), polysaccharides (PS), and bioactive lipid fractions have shown strong cardioprotective effects such as antithrombotic and anti-PAF in washed rabbit platelets stimulated by PAF and thrombin [164]. Gilthead sea bream (*Sparus aurata*) polar lipids have shown an anti-atherosclerotic effect via modulation of PAF metabolism and decreasing activity and levels of PAF in the blood [165]. Phosphatidylcholine (PC) is the most predominant phospholipid (PL) found in marine sources such as mackerel, rainbow trout, tuna, and salmon, followed by phosphatidylethanolamine (PE). Krill (*Euphausia superba*) oil (KO), a prominent source of marine PLs, show a protective effect on heart failure by reducing heart remodeling [166]. KO increases the amount of n-3 PUFA in the myocardial tissue and reduces the risk of left ventricular (LV) remodeling when taken before myocardial infarction (MI) induction [167]. It also shows a cardioprotective effect by improving blood lipids in dyslipidemia [166]. Marine carotenoids are important bioactive substances with physiological effects connected to the protection of chronic illnesses such as CVDs. These chronic illnesses, including CVDs, have been caused by oxidative stress and inflammation [168]. The dietary supplement, astaxanthin, a xanthophyll carotenoid, is an effective anti-inflammatory and antioxidant in the CVD model. Astaxanthin has been used in human clinical research to evaluate its bioavailability, safety, and clinical features related to oxidative stress and inflammation in CVDs, and no negative consequences were found [118].

Vitamins A and E are abundant in shark liver oil and possess important bio-functionalities, cardio-protective, and antioxidant effects. Their antioxidant effects protect the body against free radicals [157]. Vitamin D and its derivatives, mainly paricalcitol, have shown powerful anti-thrombotic and anti-inflammatory effects against thrombotic PAF and inflammatory-related pathways [169]. Vitamin D insufficiency has been related to greater mortality and CVD incidence via several mechanisms, including the activation of the renin-angiotensin-aldosterone system, oxidative stress, altered inflammatory pathways, and aberrant nitric oxide regulation [170]. Vitamin E has anti-inflammatory, anti-thrombotic, and antioxidant properties. Its antioxidant properties lower the incidence of CVDs by preventing LDL oxidation [157]. Vitamin E or fish oil was proven to reduce atherosclerosis in rabbits with high cholesterol [82]. Fish oil and n-3 PUFA have been adversely linked to higher levels of LDL cholesterol as well as a possible increase in LDL oxidizability owing to unfavorable lipid changes, overcome by vitamin E co-supplementation, which promotes anti-atherogenic lipid modifications and overall cardiovascular protection, including elevated HDL(2)-cholesterol levels, lowered postprandial lipemia, decreased triacylglycerol-rich lipoprotein levels, and lowered remnant levels [171].

## 6. Potential Effect of Marine Drug Targeting ROS in CVDs

Reactive oxygen species (ROS) have been generated in terms of H_2_O_2_, hydroxyl radicals, and superoxide anions (O_2_^−^) during numerous cascades of cellular processes, including mitochondria respiration. It is also involved in various biological processes, including regulating cellular homeostasis and cell signaling [112]. Researchers have already even reported a significant positive correlation between increased ROS levels and the severity of CVDs [172]. ROS have been implicated in cellular damage, apoptosis, and necrosis, as well as the direct oxidizing effect on several macromolecules such as DNA, RNA, and proteins during CVDs pathogenesis [173]. Moreover, decreased in endothelial nitric oxide synthase leads to decreased in nitric oxide (NO) production, which consequences in an increase in ROS that has been linked to endothelial damage by its interaction with other molecules to produce peroxynitrite radical in hypertension [174]. In addition, patients with systemic arterial hypertension have shown higher ROS levels and reduced NO availability [73]. Several marine drugs, including astaxanthin, fucoidan, fucoxanthin, xyloketal B, histochrome, and spirulina maxima, show potential effects via targeting ROS in CVDs. The antioxidant activity of astaxanthin has been proven in in vivo and in vitro studies via scavenging superoxide, hydroxyl radicals, and hydrogen peroxide and protection from lipid peroxidation [134]. Astaxanthin has shown antioxidant activity through inhibition of ROS generation and is effective against CVDs. A study on rabbits confirmed that it decreased non-protein thiol levels and lipid peroxidation by increasing CAT, SOD, and thioredoxin activity and inhibited ROS generation in the aortic valve [175].

Fucoidan has shown an anti-atherosclerotic effect by triggering various signaling pathways that control lipid metabolism, inhibit inflammation, and reduce oxidative stress. An in vivo study revealed that fucoidan treatment shows a preventive effect against atherosclerosis through the reduction in ROS generation and the expression of ROS generation-related proteins such as endothelial nitric oxide synthase, superoxide dismutase 1, and NADPH oxidase subunit 2/4 in the aorta of LDLR-/-mice. Fucoidan also partially recovers the lipid peroxidation and antioxidant defense system in a mouse model of alimentary hyperlipidemia [176]. Fucoxanthin is another marine drug with a powerful antioxidant effect by inhibiting ROS generation in heart valve cells. In an in vivo study on rats, fucoxanthin has shown a protective effect against H_2_O_2_-induced ROS generation through decreasing oxidative stress, promoting better cell survival, and preventing DNA damage [91]. Xyloketal B has also shown potent antioxidant activity in atherosclerotic disease through scavenging DPPH free radicals and inhibiting ROS generation induced by oxidized low-density lipoprotein (LDL). It might preserve nitric oxide bioavailability in the existence of higher ROS. It has also shown antioxidant activity in the zebrafish model and vascular endothelial cells by heme oxygenase-1 (HO-1) induction [78]. Histochrome is a bio-safe and potent agent that shows a cardioprotective effect against ROS generation or oxidative stress in human cardiac progenitor cells [112]. In the clinical trial study, *Spirulina maxima* showed a cardioprotective effect against systemic arterial hypertension after decreasing oxidative stress and endothelial damage [73].

## 7. Future Prospectus

CVDs are the leading cause of death, affecting millions of people worldwide [177]. Various drugs have been approved to treat CVDs, including antithrombotic agents, beta-blockers, diuretics, calcium channel blockers, lipid-lowering drugs, and renin-angiotensin system (RAS)-acting agents [178]. Several other new synthetics and natural herbal drugs have been recently discovered to treat CVDs globally. However, CVDs are not completely cured or eradicated by these drugs, and cases are still increasing at a high rate globally. Researchers are continuously exploring novel targets and agents that can reduce the burden of CVDs. Marine-derived drugs are emerging therapeutics in the recent era [179,180]. Experiments and investigations have indicated several marine natural products that are effective for CVDs with minimum adverse effects. Marine drugs can be used in severe conditions for multiple complications such as hypertension, atherosclerosis, myocardial infarction, etc. These drugs may decrease the severe chest pain of anginal pectoris and some other CVDs complications. In the present review, we have emphasized different marine-derived compounds such as asperlin, saringosterol, astaxanthin, manzamine A, xyloketal B, docosahexaenoic acid, echinochrome A, tetrodotoxin, botulinum toxin, zeaxanthin heneicosylate, and fucoxanthin for various CVDs. All of these marine-derived compounds have shown very encouraging results in the in vitro and in vivo studies. Moreover, thousands of marine-derived compounds are added every year to the library of marine compounds. Those compounds need to be explored for their therapeutics in CVDs. A nano-formulation-based approach must also be developed to delivered marine-based compound and are the particular target of interest to avoid side effects. Researchers may directly utilize marine-derived compounds as initial leads for the development of new medications that are more specialized than the original molecule. Based on this, it appears that marine natural products could represent a promising “library” of natural compounds for developing new therapies as adjuvants to gold standard therapies, enhancing the efficacy of conventional drugs, and exerting synergistic or additive positive effects for cardiovascular diseases.

## 8. Conclusions

Several synthetic drugs are those most often used today in pharmaceutical corporations. Pharmaceutical corporations have given up on bioactive chemical research for many years in favor of developing and manufacturing synthetic molecules. However, research into natural substances has resumed since the early 2000s and has a place within biomedical investigations. The superior biocompatibility of natural chemicals compared to manufactured pharmaceutical products, without discounting the significant variety of these molecules and their effects, are one of the primary drivers for this return to natural medicines. Marine chemicals and their derivatives have grown in pharmaceutical and medical research during the last 10 years. Unquestionably, one of the reasons is the abundance of available molecules and secondary metabolites, as well as their diversity due to the many unfavorable habitats of the seas and the virtually endless variety of creatures that inhabit them. Antioxidant and anti-inflammatory characteristics, particularly useful for treating cardiovascular disorders, are among the many pharmacological activities on which the research is focused. This review has discussed the therapeutic potential of these marine-derived compounds for CVDs with underlying mechanisms.

## Figures and Tables

**Table 1:** Classification of natural marine compounds.

**1. Based on the source**				
**(i). Marine microorganism**				
**Marine source**	**Fungus name**	**Marine drugs/metabolites**	**Fungus source Region**	**References**
A. Marine-Derived Fungi	*Arthrinium arundinis* ZSDS1-F3	4-hydroxy-2-pyridone alkaloids, arthpyrones	Xisha Islands, China	[28]
	*Stachybotrys chartarum*	Phenylspirodrimanes, named chartarlactams	Weizhou Island in Beibuwan Bay, Guangxi Province of China	
	*Aspergillus versicolor* MF359	5-methoxydihydrosterigmatocystin	Bohai Sea, China	
	*Aspergillus terreus* SCSGAF0162	Territrem and butyrolactone derivatives	South China Sea	
	*Nigrospora oryzae* SCSGAF 0111	Citrinins, nigrospins B and C	South China Sea	
B. Marine-Derived Bacteria	*Bacillus subterraneus* 1159	Bacilsubteramide A	South China Sea	[29]
	*Acinetobacter* sp. ZZ1275	Indolepyrazine A and B	Coastal area of Karachi, Sindh, Pakistan	
	*Streptomyces* sp. SCSIO 1179	Dienomycin, 6-methoxy-7′,7′′-dichlorochromopyrrolic acid	South China Sea	
	Transfected *Escherichia coli*	Tricepyridinium	Shikine-jima Island in Japan	
**(ii). Marine Invertebrates**				
A. Marine Sponges	*Psammocinia vermis*	Psammocindoles A–C	Chuja-do, Korea	[30]
	*Guitarra fimbriata* and *G. fimbriata*	Guitarrins A–E, aluminumguitarrin A	Chirpoy Island in the Pacific Ocean and Urup Island, Sea of Okhotsk	
	*Fascaplysinopsisreticulata*	(+)- and (−)-oxoaplysinopsins A–G	Xisha Island in the South China Sea	
	*Geodia barretti*	Bromooxindole, geobar-rettin A, B and C and bromoindole	West of Iceland	
B. Bryozoans	*Amathia lamourouxi*	2,5-dibromo-1-methyl-1H-indole-3-carbaldehyde	Rock pools of Woolgoolga and storm debris from Korora Beach, Coffs Harbor, New South Wales, Australia	[31]
	*Flustra foliacea*	Flustramines Q–W and flustraminols C–H	South-west coast of Iceland	
	*Securiflustra securifrons*	Securamines H–J	West Spitzbergen	
**(iii). Marine Plants**				
A. Algae	*Laurencia similis*	Brominated indoles	South China Sea	[32]
B. Mangrove Trees	*Acanthus ilicifolius* Linn	Acanthiline A	Zhanjiang Mangrove National Nature Reserve, Guangdong Province, China	
**2. Based on biological activities**				
**Biological activities**	**Marine drugs**			**References**
Antibacterial activity	Asperthins A, dienomycin, 6-methoxy-7′,7′′-dichlorochromopyrrolic acid, brominated indoles, tris-indole tulongicin A, 5-Bromotrisindoline, 6-bromotrisindoline, bis-indole alkaloid myrindole A, pyrazino bis-indole dragmacidin G, bis-indole dihydrospongotine C, spongosoritins B, spongosoritins D and spongocar-bamides A, cephalosporin C, rifamycin, okadaic acid, acanthifolicin			[32,33,34,35,36]
Antiviral activity	Fusaindoterpenes A and B, fusariumindole A and B, ascandinines C, raistrickindole A, scequinadolines A, scedapins C, dihydrospongotine C, Tulongicin A, vidarabine			[36,37,38,39,40,41]
Antifungal activity	Asperthins E, fumigatoside E, Penochalasins I and J, indolepyrazines A and B,			[33,42,43,44,45]
Antibiofilm activity	2-(2-methyl-3-en-2-yl)-1H-indole-3-carbaldehyde			[46]
Anti-inflammatory activity	Sperversamides F and G, asperversamides B and C, asperthins A, E and F, geobarrettins B and C, flustramines Q, S and U, flustraminols H, zonarol, prepacifenol, tetrado toxin			[33,47,48,49]
Antiparasitic activity	5-Bromotrisindoline, 6-bromotrisindoline, dragmacidin G			[35,50]
Analgesic activity	Ziconotide			[36,51]
Cardioprotective activity	Omega-3 acid ethyl esters, eldoisin, laminin, xyloketal B			[51,52]
Anti-Quorum sensing activity	Aspertoryadins F and G			[53]
Neuroprotective activity	16,17-dihydroxydeoxydihydroisoaustamide, 16α,17α-dihydroxydeoxydihydroisoaustamide, A-conotoxins, cembranoids, ircinialactams, dysideamine			[22,54]
Anticancer activity	Ascandinines D, Asperindoles A, penochalasins I, penochalasin K, candidusin D, dienomycin, 6-methoxy-7′,7′′-dichorochromopyrrolicacid, (+)-oxoaplysinopsin C (133a), (−)-oxoaplysinopsin C, dragmacidin G, Dragmacidins I and J, Spongosoritins A–D, cytarabine, fludarabine phosphate, nelarabine, eribulin mesylate, plitidepsin, trabectedin, brentuximab vedotin, crassian acetate, sinularin			[34,35,36,43,44,48,55,56,57]
Antidiabetic activity	Scequinadoline J, penerpenes A and B, enerpenes E, F and H, SF5280-415, psammocindoles A–C, (±)-Oxoaplysinopsin B			[30,40,58,59,60]
Pro-angiogenic activity	Dinotoamide J			[54]
Enzyme inhibitors	Guitarrins C, spongosoritins B and C, spongocarbamides A and B, xestosaprols, tasiamide B, leucettamines, petrosamine			[22,48,61]

**Table 2:** Preclinical study of marine drugs in various CVDs.

S. No.	CVDs	Marine Drug Name	Species	Dose, Route and Time	MOA	Model Inducing Agents	Outcomes/Biological Effects	References
1.	Hypertension	Protein hydrolysate *Ulva intestinalis derived peptides* FGMPLD and MELVLR	In vitro	2.5 mg/mL of each hydrolysate	Inhibit ACE	ACE-induced hypertension	Antihypertensive effect	[70]
		Wakame (Undaria pinnatifida) derived peptides (Val-Tyr, Ile-Tyr, Phe-Tyr, and Ile-Trp)	Rats	1 mg/kg	Inhibit ACE	Spontaneously hypertensive rats	Antihypertensive effect	[69]
		Low molecular mass potassium alginate (L-PA)	Rats	250, 500 mg/kg, once orally for 30 days	Increased the excretion of sodium salt	Deoxycorticosterone acetate (DOCA)-salt-induced hypertension	Antihypertensive effect	[75]
		Alginate oligosaccharides (AOS)	Rats	5, 10 and 20 mg/kg for 5 weeks	Suppressed intestinal absorption of salts leads to vasodilatory effect	Monocrotaline (MCT)-induced pulmonary hypertension	Decrease P-selectin expression in serum, pulmonary tissue, and pulmonary arteries	[71]
		*Gracilariopsis lemaneiformis* derived Peptides (FQIN [M(O)] CILR and TGAPCR)		10 mg/kg, orally for 24 hrs.	Inhibit angiotensin-converting enzyme (ACE)	ACE-induced hypertension	Antihypertensive effects, reduced both systolic and diastolic blood pressure	[68,76]
		Xyloketal B		20 mg/kg/day, 20 for 12 weeks	Promoted endothelial NO release and protected against atherosclerosis through the Akt/eNOS pathway.	Phenylephrine (Phe)-induced contractions cause hypertension	Antihypertensive effect, Decrease the systolic and diastolic blood pressure, vasorelaxant effect, anti-inflammatory and anti-atherosclerotic effects	[51]
2.	Atherosclerosis	Asperlin	Mice	80 mg/kg/day, orally for 12 weeks	Inhibit the pro-inflammatory markers	In vitro (LPS-induced foam cell formation in macrophages) and in vivo (high-fat diet-induced-atherosclerosis lesion in ApoE^−/−^ mice)	Athero-protection via decreasing the expression levels of iNOS, IL-1β, and TNF-α, and increased the expression of IL-10 and IL-4,	[77]
		Xyloketal B		7, 14 and 28 mg/kg/day, orally for 16 weeks	Inhibit the oxidative endothelial dysfunction and increase nitric oxide (NO) bioavailability	High-fat diet-induced atherosclerotic lesion	Strong antioxidant actions, reduced the levels of vascular oxidative stress, improving the impaired endothelium integrity and NO-dependent aortic vasorelaxation in atherosclerotic	[78]
		Saringosterol	Mice	50 mg/kg/day, orally for 2 weeks	Altered the liver X receptor (LXR)-regulated gene expression	High-fat diet-induced atherosclerosis	Decrease cholesterol level and anti-atherogenic effect	[79]
		Manzamine	ApoE-/- deficient mice	30 mg/kg/day, orally for 80 days	Inhibited the acyl-CoA: cholesterol acyl-transferase (ACAT) activity		Decrease the level of total, free and LDL-cholesterol, and triglycerides	[80]
		Astaxanthin	ApoE-/- deficient mice	0.03% (equivalent to approx. 200 mg/day in humans), orally for 4 weeks	By increasing the expression of LDL receptor (LDLR)	High-fat diet (high fat 15% and high cholesterol 0.2%)-induced atherosclerosis	Decrease the level of total triglyceride, and cholesterol	[81]
		Vitamin E	Rabbit	450 mg/1000 g chow fed orally for 6-weeks	Decrease creatine kinase elevation	High cholesterol-enriched diet induced atherosclerosis	Lowered aortic TBARS levels, favorable prostanoid generation, and diminished atherosclerotic lesions	[82]
		Fascaplysin	BALB/c mice	5 mg/kg, intraperitoneally 19 h and 1 h before inducing thrombus	Inhibited kinase enzyme, and decreased GPIIb/IIIa activation	Photochemical-induced thrombus	Anti-platelet, and anti-thrombus effect via inhibiting GPIIb/IIIa integrin complex	[83]
		Isaridin E	C57BL/6J mice	12.5, 25, 50 and 100 mg/kg, orally at 1, 24 and 48 h before FeCl_3_-Induced thrombus	Inhibited adenosine diphosphate	FeCl_3_-induced thrombus	Antithrombotic, and antiplatelet effect in atherosclerosis	[84]
3.	Myocardial Infarction (MI)	Cyanobacterial extract (CBE) and CBE+ GNPs	Rats	200 mg/kg/day, intraperitoneally for 14 days	Inhibit the depletion of the anti-oxidant enzymes (GRx and SOD)	Isoproterenol-induced MI	Decrease ST and QT segments, heart rate, and serum activities of creatine phosphokinase (CPK), reduced systolic and diastolic blood pressure	[85]
		Docosahexaenoic acid (DHA)	Pig	45 mg or 1 mg/kg, infused in pericardial space for 40 min.	Inhibited Ca^2+^ and Na^+^/Ca^2+^ exchanger currents and prevented intracellularly Ca^2+^ concentration	Sternotomy method was used to expose the heart and induce MI	Decrease fatal arrhythmias and infarct sizes, decrease heart rates and reduce ventricular arrhythmia scores during ischemia.	[86,87]
4.	Cardiac Stroke	Xyloketal B	Mice	50 mg/kg intraperitoneally 0, 1 and 2 h. after ischemia	By suppressing TLR4/NF-κB/ROS signaling pathway	Transient middle cerebral artery occlusion-induced stroke	Decrease ROS production, focal cerebral ischemia, and reduce infarction volume.	[88]
5.	Cardiac Arrythmia	Botulinum toxin-chitosan nanoparticles (BTN)	Rat	5 U/kg, subepicardial injection for 14 days	Decreased the activation of Ca^2+^, K^+^ and Na^+^ channels	Calcium chloride-, barium chloride- and electrically induced arrhythmia	Inhibit ventricular fibrillation, reduce the incidence of ventricular arrhythmias	[89]
		Eicosapentaenoic acid (EPA)	Dog	5–15 μmol/L, intravenous infusion for 50–60 min.	Inhibition of Ca^2+^ and Na^+^/Ca^2+^ exchanger currents increase Ca^2+^ concentrations intracellularly	High Ca^2+^, ouabain, lysophosphatidylcholine, acylcarnitine, β-adrenergic agonist, and Ca^2+^ ionophore-induced arrhythmia	Inhibit cardiac arrhythmia through inhibition of fatal ischemia, prevents tachyarrhythmias	[86,90]
6.	Heart value disease	Fucoxanthin (Fx)	Dog	60 mg/kg twice daily for 2 years	Reduced oxidative stress-induced DNA damage	H_2_O_2_-induced oxidative stress-induced heart value damages	Strong antioxidant, anti-inflammatory, and antitumor properties, improved cell survival and, protective effect against calcification	[91]
7.	Cardiac dysfunction	Zeaxanthin (ZH)	Rats	250 μg/kg, orally for 4 weeks	Elevated retinoid receptor alpha (RAR-α) expression in cardiac tissue	d-galactose-induced cardiac dysfunction	Improve serum levels of homocysteine, creatinine kinase isoenzyme and lactate dehydrogenase, increase the cardiac contents of glucose transporter-4 and superoxide dismutase, decrease inducible nitric oxide synthetase and interleukin-6	[92]

**Table 3:** Marine drugs class, source, and their biological effects in CVDs.

Class	Marine Drugs	Marine Source	Biological Effects	References
Pigments (Xanthophyll carotenoid)	Astaxanthin	Microalgae (*Haematococcus pluvialis, Chlorella zofingiensis,* and *Chlorococcum* sp.), fungi (red yeast *Phaffia rhodozyma*) crustacean, Shrimp, lobster, trout, krill, salmon, fungi, complex plants, seafood, flamingos, and quail	Cardioprotective (atherosclerosis protective), antidepressant, antioxidant, anti-inflammatory, neuroprotective, anticancer, antidiabetic, gastrointestinal protective, and hepatoprotective.	[22,134,135,136,137]
	Fucoxanthin	Macroalgae (*Undaria pinnatifida, Hijikia fusiformis* and *Sargassum fulvelum*)	Cardioprotective, Antioxidant, thermogenesis, stroke prevention, anti-inflammatory, anticancer, and improved blood pressure and liver function.	[118]
Soluble dietary fibers	Alginate/Alginic acid	Brown macroalgae (*Pseudomonas* and *Azotobacter, Pseudomonas aeruginosa, Azotobacter chroococcum*)	Cardioprotective (used in myocardial infarction), antimicrobial, anti-inflammatory, anticancer, and antidiabetic.	[137,138,139,140]
	Carrageenan	Red macroalgae *Chondrus armatus* (Gigartinaceae), *Eucheuma, Betaphycus, Kappaphycus,* and *Chondrus crispus*	Cardioprotective (used for ischemic heart disease), immunomodulator, anti-hypercholesterolaemic, anti-inflammatory, anticancer, and antivirus properties.	[141]
	Agar	*Gelidium, Pterocladia*, and *Gracilaria gracilis* (Rhodophyta)	Cardioprotective, anticoagulant, antiviral, antioxidative, anticancer, and immune-modulating activities.	[137,138,142]
	Fucoidans	*Fucus vesiculosus and L. japonica*	Cardioprotective, coagulant activity.	
	Ulvans	*Ulva pertusua*	Anti-oxidant activity.	
Peptides	Leu-Lys-Gln-Glu-Leu-Glu-Asp-Leu-Leu-Glu- Lys-Gln-Glu	*Crassostrea gigas*	Anticancer, antihypertensive, anti-thrombosis, antioxidant, and anticoagulant properties.	[137,138]
	Pepsin-hydrolyzed peptide (VECYGPNRPQF)	Seaweed (*Chlorella vulgaris*)	Potent antioxidant, anticancer, opioid agonists or antagonists, immunomodulatory, antithrombotic, anti-atherosclerotic, and antimicrobial activities.	[143]
	Antitumor polypeptide Y2	*Spirulina platensis*		
	Phycobili protein byproduct	*Porphyra columbina*	Immunosuppressive effects through increasing IL-10 production and preventing the production of IFN-γ and TNF-α.	[144]
	Leu-Trp, Val-Tyr, Ile-Tyr, Phe-Tyr, and Ile-Tyr	*U. pinnatifida*	Antihypertensive effects.	[69]
	α and β subunits of phycoerythrin	Red seaweed (*P. palmate*)	ACE inhibition activity.	[145]
	Ile-Leu-Ala-Pro, Leu-Leu-Ala-Pro, and Met-Ala-Gly-Val-Asp-His-Ile	Macroalga (*Palmaria palmata*)	Inhibited DPP-IV (ischemic cardiovascular disease marker).	[146]
	Ile-Pro and Ala-Phe-Leu	*Chlorophyta U. rigida*	ACE inhibition activity.	[76]
Phlorotannins (phenolic compounds)	Phloroglucinol	*Hyaleucerea fusiformis*	Potent antioxidant effects, anti-inflammatory and anticancer effects, inhibit the hyaluronidase enzyme.	[137,138,147]
	Phlorofucofuroeckol A	*Eisenia bicyclis, Ecklonia cava* (brown algae)	Antidiabetic, antihypertensive, antioxidant activity.	
Minerals	Na, K, Mg, P, I, Zn, and Fe	Microalgae (*Chlorococcum humicola* and *Chlorella vulgaris*)	Used for the prevention and treatment of CVDs.	[137,138]
	Na^+^/K^+^ ratio, Mg		Controls blood pressure, prevent metabolic syndrome and atherosclerosis.	
	NaCl		Increases arterial constriction and peripheral vascular resistance, increased blood pressure.	
	K^+^		Decreases the blood pressure, preventing problems associated with high blood pressure.	
Lipids	Eicosapentanoic acid	Microalga * Nannochloropsis gaditana * (NG)	Reduced inflammatory genes expression and inhibits platelets.	[138,148]
	Arachidonic acid	*Mortierella alpina* (saprophytic, oleaginous soil fungus)	Activates the immune functions, pro-inflammatory properties, maintaining homeostasis, anticancer, cardioprotective, anti-psoriasis, anti-arteriosclerosis, and antiulcer properties.	[138,149]
Sulphated fucans	Fucoidan	Brown seaweeds (*Sargassum ilicifolium* and *Sargassum angustifolium*)	Reduces lipid deposition in atherosclerosis, hypolipidemic effect controls obesity. CVDs	[150,151]
Marine Neurotoxins	Tetrodotoxin (TTX)	Sea-slug * Pleurobranchaea maculata * and pufferfish * Takifugu niphobles *	Visceral analgesic, local anesthetic, controls cardiac contractions.	[124,152,153,154]
Non-peptide neurotoxin	Saxitoxin (STX)	Dinoflagellates species from the genera * Alexandrium, Gymnodinium, Centrodinium * and * Pyrodinium *	Wound healing, corneal analgesic, controls myocardial impulse generation.	[124,154,155]
Fungus	Xyloketal B	Mangrove fungus *xylaria* species		[156]

**Table 4:** List of marine drugs under clinical trials.

Sr. No.	Marine Drugs	Disease	Sponsor (Organization)	Phase (Number of Participants)	Duration of Intervention	Type of Study	Current Status	Possible MOA	Measured Outcome	NCT
1.	**Astaxanthin** + omega 3 fatty acids + vitamin E + hawthorn (Ritmonutra)	Arrhythmia	IRCCS Policlinico S. Donato	NA (24)	Daily 2 tablets, orally for 4 weeks	Interventional	Unknown	Decrease the number of SVEB	Decrease SVEB-related symptoms via a symptom score and QOL survey	NCT02087033
2.	**Astaxanthin** + monacolin K + berberine + policosanol + folic acid + coenzyme Q10 (Nutraceutical combination)	Atherosclerosis	University Of Perugia	4 (26)	Daily one pill for 3 months	Interventional	Completed (Phase 4)	Decrease the lipid profile	Changes in LDL-C, PCSK9, hs-CRP levels, and arterial stiffness	NCT03470376
3.	**Astaxanthin** + omega-3 polyunsaturated fatty acids + vitamin E + vitamin B complex + hawthorn + diet (Ritmonutra)	Benign ventricular and supraventricular arrhythmias	Federico II University	4 (1500)	4 weeks	Interventional	Completed (Phase 4)	Regulate cardiac pacing, lowering the overall incidence of BES and enhancing QOL	Reduced ventricular and atrial arrhythmias and improved QOL	NCT01647984
4.	**Alginate** beverage	Cardiovascular Disease	University of Copenhagen	NA (96)	Daily 3 × 500 mL for 12 weeks	Interventional	Completed	Decrease body weight and major risk markers of CVDs	Improvement in body weight, blood pressure, risk markers for CVDs and T2D	NCT01231178
5.	**Alginate** Hydrogel	Heart Failure	Xijing Hospital	NA (10)	Single-use implanted in the myocardium	Interventional	Enrolling by invitation	Reduce the symptoms of left ventricular ischemia and non-ischemic cardiomyopathy	The device is successfully setup, reaches the target location, and the occurrence rate of SADE	NCT04781660
6.	Sodium **Alginate** Calcium Gluconate (IK-5001)	Acute MI, CHF, ST-elevation MI	Bellerophon BCM LLC	NA (303)	4 mL via intracoronary slow bolus injection for 15 to 30 s after 2 days PCI and 5 days symptoms	Interventional	Completed	Decrease ST segment, and prevent ventricular remodeling and CHF in MI	Assessed LVEDVI by echocardiography, a six-minute walk test performed, and alginate measured in plasma and urine	NCT01226563
7.	AMR101 (ethyl ester of **eicosapentaenoic acid**)	Cardiovascular diseases	Amarin Pharma Inc.	3 (8179)	Daily for 28 days	Interventional	Completed (Phase 3)	Decreased CVDs’ events by reducing triglycerides	Measured CVD death, nonfatal MI, nonfatal stroke, coronary revascularization, or unstable angina by invasive/non-invasive testing	NCT01492361
8.	Icosapent ethyl (ethyl ester of **eicosapentaenoic acid**)	CVDs, atherosclerotic CVD, MI, and CHF	Canadian Medical and Surgical Knowledge Translation Research Group	NA (200)	8 weeks	Observational	Active, not recruiting	Decrease LDL and hypertriglyceridemia	The demographic and biochemical data are consistent with the cohort’s REDUCE-IT baseline requirements	NCT05271591
9.	**Omega-3 polyunsaturated fatty acids**	Cardiovascular disease	Laval University	NA (200)	Daily 3 g for 6 weeks	Interventional	Active, not recruiting (NA)	Genetic polymorphisms within genes functioning as fatty acids sensors affect the alterations in metabolic risk factors caused by n-3 PUFAs	Changes in blood lipids, blood pressure, anthropometric measures, plasma glycemia, insulin levels, and gene expression levels	NCT01343342

NA: Not applicable; QOL: Quality of life; SVEB: Supraventricular ectopic beats; PCSK9: Proprotein convertase subtilisin/Kexin type 9; hsCRP: High-sensitivity C-reactive protein; BES: Benign extrasystoles; T2D: Type 2 diabetes; SADE: Serious adverse device events; LVEDVI: Assessed left ventricular end-diastolic volume index; n-3 PUFAs: Omega-3 polyunsaturated fatty acids.

## Data Availability

Not applicable.

## References

[1] Xiang Z., Han M., Zhang H. (2022). Nanomaterials Based Flexible Devices for Monitoring and Treatment of Cardiovascular Diseases (CVDs). Nano Res..

[2] Roth G.A., Mensah G.A., Johnson C.O., Addolorato G., Ammirati E., Baddour L.M., Barengo N.C., Beaton A., Benjamin E.J., Benziger C.P. (2020). Global Burden of Cardiovascular Diseases and Risk Factors, 1990–2019: Update From the GBD 2019 Study. J. Am. Coll. Cardiol..

[3] Abd M., Mohammed E., Badawy D., Naing L., Johar S., Ong S., Rahman H.A., Lin C., Raja C., Pengiran I. (2022). Scoping Review: Are CVDs Risk Calculators Using the Digital Platform Benecial for CVDs Prevention and Management?. Res. Sq..

[4] Roth G.A., Mensah G.A., Fuster V. (2020). The Global Burden of Cardiovascular Diseases and Risks: A Compass for Global Action. J. Am. Coll. Cardiol..

[5] Woodward M. (2019). Cardiovascular Disease and the Female Disadvantage. Int. J. Environ. Res. Public Health.

[6] Vogel B., Acevedo M., Appelman Y., Bairey Merz C.N., Chieffo A., Figtree G.A., Guerrero M., Kunadian V., Lam C.S.P., Maas A.H.E.M. (2021). The Lancet Women and Cardiovascular Disease Commission: Reducing the Global Burden by 2030. Lancet.

[7] Fuchs F.D., Whelton P.K. (2020). High Blood Pressure and Cardiovascular Disease. Hypertension.

[8] Dabravolski S.A., Sukhorukov V.N., Kalmykov V.A., Orekhov N.A., Grechko A.V., Orekhov A.N. (2022). Heat Shock Protein 90 as Therapeutic Target for CVDs and Heart Ageing. Int. J. Mol. Sci..

[9] Valipour M., Irannejad H., Emami S. (2022). Papaverine, a Promising Therapeutic Agent for the Treatment of COVID-19 Patients with Underlying Cardiovascular Diseases (CVDs). Drug Dev. Res..

[10] Ho C.K., Kleeff J., Friess H., Büchler M.W. (2005). Complications of Pancreatic Surgery. HPB.

[11] Jankowski J., Floege J., Fliser D., Böhm M., Marx N. (2021). Cardiovascular Disease in Chronic Kidney Disease Pathophysiological Insights and Therapeutic Options. Circulation.

[12] Festa M., Sansone C., Brunet C., Crocetta F., Di Paola L., Lombardo M., Bruno A., Noonan D.M., Albini A. (2020). Cardiovascular Active Peptides of Marine Origin with ACE Inhibitory Activities: Potential Role as Anti-Hypertensive Drugs and in Prevention of SARSCoV-2 Infection. Int. J. Mol. Sci..

[13] Ferraz C.A.A., Grougnet R., Nicolau E., Picot L., de Oliveira Junior R.G. (2022). Carotenoids from Marine Microalgae as Antimelanoma Agents. Mar. Drugs.

[14] Zhou J.-B., Luo R., Zheng Y.-L., Pang J.-Y. (2017). Recent Advances in the Discovery and Development of Marine Natural Products with Cardiovascular Pharmacological Effects. Mini-Rev. Med. Chem..

[15] Liang B., Cai X.-Y., Gu N. (2021). Marine Natural Products and Coronary Artery Disease. Front. Cardiovasc. Med..

[16] Newman D.J., Cragg G.M. (2016). Natural Products as Sources of New Drugs from 1981 to 2014. J. Nat. Prod..

[17] Jiménez C. (2018). Marine Natural Products in Medicinal Chemistry. ACS Med. Chem. Lett..

[18] Raven P.H., Margulis L., Schwartz K.V. (1988). Five Kingdoms: An Illustrated Guide to the Phyla of Life on Earth. Bryologist.

[19] Donia M., Hamann M.T. (2003). Marine Natural Products and Their Potential Applications as Anti-Infective Agents. Lancet Infect. Dis..

[20] Malve H. (2016). Exploring the Ocean for New Drug Developments: Marine Pharmacology. J. Pharm. Bioallied Sci..

[21] Khalifa S.A.M., Elias N., Farag M.A., Chen L., Saeed A., Hegazy M.E.F., Moustafa M.S., El-Wahed A.A., Al-Mousawi S.M., Musharraf S.G. (2019). Marine Natural Products: A Source of Novel Anticancer Drugs. Mar. Drugs.

[22] Catanesi M., Caioni G., Castelli V., Benedetti E., D’angelo M., Cimini A. (2021). Benefits under the Sea: The Role of Marine Compounds in Neurodegenerative Disorders. Mar. Drugs.

[23] Voultsiadou Eleni E. (2010). Therapeutic Properties and Uses of Marine Invertebrates in the Ancient Greek World and Early Byzantium. J. Ethnopharmacol..

[24] Fu X.M., Zhang M.Q., Shao C.L., Li G.Q., Bai H., Dai G.L., Chen Q.W., Kong W., Fu X.J., Wang C.Y. (2016). Chinese Marine Materia Medica Resources: Status and Potential. Mar. Drugs.

[25] Pohnert G., Schulz S. (2004). Chemical Defense Strategies of Marine Organisms. The Chemistry of Pheromones and Other Semiochemicals I.

[26] Ahmed I., Asgher M., Sher F., Hussain S.M., Nazish N., Joshi N., Sharma A., Parra-Saldívar R., Bilal M., Iqbal H.M.N. (2022). Exploring Marine as a Rich Source of Bioactive Peptides: Challenges and Opportunities from Marine Pharmacology. Mar. Drugs.

[27] Nigam M., Suleria H.A.R., Farzaei M.H., Mishra A.P. (2019). Marine Anticancer Drugs and Their Relevant Targets: A Treasure from the Ocean. DARU, J. Pharm. Sci..

[28] Liming J., Chunshan Q., Xiyan H., Shengdi F. (2016). Potential Pharmacological Resources: Natural Bioactive Compounds from Marine-Derived Fungi. Mar. Drugs.

[29] Wibowo J.T., Ahmadi P., Rahmawati S.I., Bayu A., Putra M.Y., Kijjoa A. (2022). Marine-Derived Indole Alkaloids and Their Biological and Pharmacological Activities. Mar. Drugs.

[30] Kwon O.S., Ahn S., Jeon J.E., Park I.G., Won T.H., Sim C.J., Park H.G., Oh D.C., Oh K.B., Noh M. (2021). Psammocindoles A-C: Isolation, Synthesis, and Bioactivity of Indole-γ-Lactams from the Sponge Psammocinia Vermis. Org. Lett..

[31] Kleks G., Holland D.C., Kennedy E.K., Avery V.M., Carroll A.R. (2020). Antiplasmodial Alkaloids from the Australian Bryozoan Amathia Lamourouxi. J. Nat. Prod..

[32] Li M.C., Sun W.S., Cheng W., Liu D., Liang H., Zhang Q.Y., Lin W.H. (2016). Four New Minor Brominated Indole Related Alkaloids with Antibacterial Activities from Laurencia Similis. Bioorganic Med. Chem. Lett..

[33] Yang J., Gong L., Guo M., Jiang Y., Ding Y., Wang Z., Xin X., An F. (2021). Bioactive Indole Diketopiperazine Alkaloids from the Marine Endophytic Fungus *Aspergillus* sp. YJ191021. Mar. Drugs.

[34] Song Y., Yang J., Yu J., Li J., Yuan J., Wong N.K., Ju J. (2020). Chlorinated Bis-Indole Alkaloids from Deep-Sea Derived *Streptomyces* sp. SCSIO 11791 with Antibacterial and Cytotoxic Activities. J. Antibiot. (Tokyo).

[35] Wright A.E., Killday K.B., Chakrabarti D., Guzmán E.A., Harmody D., McCarthy P.J., Pitts T., Pomponi S.A., Reed J.K., Roberts B.F. (2017). Dragmacidin G, a Bioactive Bis-Indole Alkaloid from a Deep-Water Sponge of the Genus Spongosorites. Mar. Drugs.

[36] Chen G., Seukep A.J., Guo M. (2020). Recent Advances in Molecular Docking for the Research and Discovery of Potential Marine Drugs. Mar. Drugs.

[37] Guo Y.W., Liu X.J., Yuan J., Li H.J., Mahmud T., Hong M.J., Yu J.C., Lan W.J. (2020). L-Tryptophan Induces a Marine-Derived *Fusarium* sp. to Produce Indole Alkaloids with Activity against the Zika Virus. J. Nat. Prod..

[38] Zhou G., Sun C., Hou X., Che Q., Zhang G., Gu Q., Liu C., Zhu T., Li D. (2021). Ascandinines A-D, Indole Diterpenoids, from the Sponge-Derived Fungus *Aspergillus candidus* HDN15-152. J. Org. Chem..

[39] Li J., Hu Y., Hao X., Tan J., Li F., Qiao X., Chen S., Xiao C., Chen M., Peng Z. (2019). Raistrickindole A, an Anti-HCV Oxazinoindole Alkaloid from Penicillium Raistrickii IMB17-034. J. Nat. Prod..

[40] Huang L.H., Xu M.Y., Li H.J., Li J.Q., Chen Y.X., Ma W.Z., Li Y.P., Xu J., Yang D.P., Lan W.J. (2017). Amino Acid-Directed Strategy for Inducing the Marine-Derived Fungus Scedosporium Apiospermum F41-1 to Maximize Alkaloid Diversity. Org. Lett..

[41] Liu H.B., Lauro G., O’Connor R.D., Lohith K., Kelly M., Colin P., Bifulco G., Bewley C.A. (2017). Tulongicin, an Antibacterial Tri-Indole Alkaloid from a Deep-Water *Topsentia* sp. Sponge. J. Nat. Prod..

[42] Limbadri S., Luo X., Lin X., Liao S., Wang J., Zhou X., Yang B., Liu Y. (2018). Bioactive Novel Indole Alkaloids and Steroids from Deep Sea-Derived Fungus *Aspergillus fumigatus* SCSIO 41012. Molecules.

[43] Huang S., Chen H., Li W., Zhu X., Ding W., Li C. (2016). Bioactive Chaetoglobosins from the Mangrove Endophytic Fungus Penicillium Chrysogenum. Mar. Drugs.

[44] Zhu X., Zhou D., Liang F., Wu Z., She Z., Li C. (2017). Penochalasin K, a New Unusual Chaetoglobosin from the Mangrove Endophytic Fungus Penicillium Chrysogenum V11 and Its Effective Semi-Synthesis. Fitoterapia.

[45] Anjum K., Kaleem S., Yi W., Zheng G., Lian X., Zhang Z. (2019). Novel Antimicrobial Indolepyrazines A and B from the Marine-Associated *Acinetobacter* sp. ZZ1275. Mar. Drugs.

[46] May Zin W.W., Buttachon S., Dethoup T., Pereira J.A., Gales L., Inácio Â., Costa P.M., Lee M., Sekeroglu N., Silva A.M.S. (2017). Antibacterial and Antibiofilm Activities of the Metabolites Isolated from the Culture of the Mangrove-Derived Endophytic Fungus Eurotium Chevalieri KUFA 0006. Phytochemistry.

[47] Li H., Sun W., Deng M., Zhou Q., Wang J., Liu J., Chen C., Qi C., Luo Z., Xue Y. (2018). Asperversiamides, Linearly Fused Prenylated Indole Alkaloids from the Marine-Derived Fungus *Aspergillus versicolor*. J. Org. Chem..

[48] Park J.S., Cho E., Hwang J.Y., Park S.C., Chung B., Kwon O.S., Sim C.J., Oh D.C., Oh K.B., Shin J. (2021). Bioactive Bis(Indole) Alkaloids from a *Spongosorites* sp. Sponge. Mar. Drugs.

[49] Di X., Wang S., Oskarsson J.T., Rouger C., Tasdemir D., Hardardottir I., Freysdottir J., Wang X., Molinski T.F., Omarsdottir S. (2020). Bromotryptamine and Imidazole Alkaloids with Anti-Inflammatory Activity from the Bryozoan Flustra Foliacea. J. Nat. Prod..

[50] El-Hawary S.S., Sayed A.M., Mohammed R., Hassan H.M., Rateb M.E., Amin E., Mohammed T.A., El-Mesery M., Muhsinah A.B., Alsayari A. (2019). Bioactive Brominated Oxindole Alkaloids from the Red Sea Sponge Callyspongia Siphonella. Mar. Drugs.

[51] Zhao L.Y., Li J., Huang X.Q., Wang G.H., Lv X.F., Meng W.F., Chen W.L., Pang J.Y., Lin Y.C., Sun H.S. (2018). Xyloketal B Exerts Antihypertensive Effect in Renovascular Hypertensive Rats via the NO-SGC-CGMP Pathway and Calcium Signaling. Acta Pharmacol. Sin..

[52] Marchioli R. (2010). Uses and Benefits of Omega-3 Ethyl Esters in Patients with Cardiovascular Disease. J. Multidiscip. Healthc..

[53] Kong F.D., Zhang S.L., Zhou S.Q., Ma Q.Y., Xie Q.Y., Chen J.P., Li J.H., Zhou L.M., Yuan J.Z., Hu Z. (2019). Quinazoline-Containing Indole Alkaloids from the Marine-Derived Fungus *Aspergillus* sp. HNMF114. J. Nat. Prod..

[54] Li P., Zhang M., Li H., Wang R., Hou H., Li X., Liu K., Chen H. (2021). New Prenylated Indole Homodimeric and Pteridine Alkaloids from the Marine-Derived Fungus *Aspergillus austroafricanus* Y32-2. Mar. Drugs.

[55] Ivanets E.V., Yurchenko A.N., Smetanina O.F., Rasin A.B., Zhuravleva O.I., Pivkin M.V., Popov R.S., Von Amsberg G., Afiyatullov S.S., Dyshlovoy S.A. (2018). Asperindoles A–D and a p-Terphenyl Derivative from the Ascidian-Derived Fungus *Aspergillus* sp. KMM 4676. Mar. Drugs.

[56] Buttachon S., Ramos A.A., Inácio Â., Dethoup T., Gales L., Lee M., Costa P.M., Silva A.M.S., Sekeroglu N., Rocha E. (2018). Bis-Indolyl Benzenoids, Hydroxypyrrolidine Derivatives and Other Constituents from Cultures of the Marine Sponge-Associated Fungus *Aspergillus candidus* KUFA0062. Mar. Drugs.

[57] Cruz P.G., Martínez Leal J.F., Daranas A.H., Pérez M., Cuevas C. (2018). On the Mechanism of Action of Dragmacidins i and J, Two New Representatives of a New Class of Protein Phosphatase 1 and 2A Inhibitors. ACS Omega.

[58] Kong F.D., Fan P., Zhou L.M., Ma Q.Y., Xie Q.Y., Zheng H.Z., Zheng Z.H., Zhang R.S., Yuan J.Z., Dai H.F. (2019). Penerpenes A-D, Four Indole Terpenoids with Potent Protein Tyrosine Phosphatase Inhibitory Activity from the Marine-Derived Fungus *Penicillium* sp. KFD28. Org. Lett..

[59] Zhou L.M., Kong F.D., Fan P., Ma Q.Y., Xie Q.Y., Li J.H., Zheng H.Z., Zheng Z.H., Yuan J.Z., Dai H.F. (2019). Indole-Diterpenoids with Protein Tyrosine Phosphatase Inhibitory Activities from the Marine-Derived Fungus *Penicillium* sp. KFD28. J. Nat. Prod..

[60] Cho K.H., Sohn J.H., Oh H. (2018). Isolation and Structure Determination of a New Diketopiperazine Dimer from Marine-Derived Fungus *Aspergillus* sp. SF-5280. Nat. Prod. Res..

[61] Guzii A.G., Makarieva T.N., Denisenko V.A., Gerasimenko A.V., Udovenko A.A., Popov R.S., Dmitrenok P.S., Golotin V.A., Fedorov S.N., Grebnev B.B. (2019). Guitarrins A–E and Aluminumguitarrin A: 5-Azaindoles from the Northwestern Pacific Marine Sponge Guitarra Fimbriata. J. Nat. Prod..

[62] Zhou B., Perel P., Mensah G.A., Ezzati M. (2021). Global Epidemiology, Health Burden and Effective Interventions for Elevated Blood Pressure and Hypertension. Nat. Rev. Cardiol..

[63] Zhou B., Carrillo-Larco R.M., Danaei G., Riley L.M., Paciorek C.J., Stevens G.A., Gregg E.W., Bennett J.E., Solomon B., Singleton R.K. (2021). Worldwide Trends in Hypertension Prevalence and Progress in Treatment and Control from 1990 to 2019: A Pooled Analysis of 1201 Population-Representative Studies with 104 Million Participants. Lancet.

[64] Erdmann K., Cheung B.W.Y., Schröder H. (2008). The Possible Roles of Food-Derived Bioactive Peptides in Reducing the Risk of Cardiovascular Disease. J. Nutr. Biochem..

[65] Yokoyama K., Chiba H., Yoshikawa M. (1992). Peptide Inhibitors for Angiotensin I-Converting Enzyme from Thermolysin Digest of Dried Bonito. Biosci. Biotechnol. Biochem..

[66] Wijesekara I., Kim S.K. (2010). Angiotensin-I-Converting Enzyme (ACE) Inhibitors from Marine Resources: Prospects in the Pharmaceutical Industry. Mar. Drugs.

[67] Abachi S., Bazinet L., Beaulieu L. (2019). Antihypertensive and Angiotensin-i-Converting Enzyme (ACE)-Inhibitory Peptides from Fish as Potential Cardioprotective Compounds. Mar. Drugs.

[68] Deng Z., Liu Y., Wang J., Wu S., Geng L., Sui Z., Zhang Q. (2018). Antihypertensive Effects of Two Novel Angiotensin I-Converting Enzyme (Ace) Inhibitory Peptides from Gracilariopsis Lemaneiformis (Rhodophyta) in Spontaneously Hypertensive Rats (SHRs). Mar. Drugs.

[69] Sato M., Hosokawa T., Yamaguchi T., Nakano T., Muramoto K., Kahara T., Funayama K., Kobayashi A., Nakano T. (2002). Angiotensin I-Converting Enzyme Inhibitory Peptides Derived from Wakame (Undaria Pinnatifida) and Their Antihypertensive Effect in Spontaneously Hypertensive Rats. J. Agric. Food Chem..

[70] Sun S., Xu X., Sun X., Zhang X., Chen X., Xu N. (2019). Preparation and Identification of ACE Inhibitory Peptides from the Marine Macroalga Ulva Intestinalis. Mar. Drugs.

[71] Hu Y., Feng Z., Feng W., Hu T., Guan H., Mao Y. (2019). AOS Ameliorates Monocrotaline-Induced Pulmonary Hypertension by Restraining the Activation of P-Selectin/P38MAPK/NF-ΚB Pathway in Rats. Biomed. Pharmacother..

[72] Shen T., Xing G., Zhu J., Zhang S., Cai Y., Li D., Xu G., Xing E., Rao J., Shi R. (2017). Effects of 12-Week Supplementation of Marine Omega-3 PUFA-Based Formulation Omega3Q10 in Older Adults with Prehypertension and/or Elevated Blood Cholesterol. Lipids Health Dis..

[73] Martínez-Sámano J., De Oca A.T.M., O.-Bocardo O.I.L., Torres-Durán P.V., Juárez-Oropeza M.A. (2018). Spirulina Maxima Decreases Endothelial Damage and Oxidative Stress Indicators in Patients with Systemic Arterial Hypertension: Results from Exploratory Controlled Clinical Trial. Mar. Drugs.

[74] Nuhu A.A. (2013). Spirulina (Arthrospira): An Important Source of Nutritional and Medicinal Compounds. J. Mar. Biol..

[75] Chen Y.Y., Ji W., Du J.R., Yu D.K., He Y., Yu C.X., Li D.S., Zhao C.-Y., Qiao K. (2010). yun Preventive Effects of Low Molecular Mass Potassium Alginate Extracted from Brown Algae on DOCA Salt-Induced Hypertension in Rats. Biomed. Pharmacother..

[76] Cho C.H., Lu Y.A., Kim M.Y., Jeon Y.J., Lee S.H. (2022). Therapeutic Potential of Seaweed–Derived Bioactive Compounds for Cardiovascular Disease Treatment. Appl. Sci..

[77] Zhou Y., Chen R., Liu D., Wu C., Guo P., Lin W. (2017). Asperlin Inhibits LPS-Evoked Foam Cell Formation and Prevents Atherosclerosis in ApoE−/− Mice. Mar. Drugs.

[78] Zhao L.Y., Li J., Yuan F., Li M., Zhang Q., Huang Y.Y., Pang J.Y., Zhang B., Sun F.Y., Sun H.S. (2015). Xyloketal B Attenuates Atherosclerotic Plaque Formation and Endothelial Dysfunction in Apolipoprotein E Deficient Mice. Mar. Drugs.

[79] Yan Y., Niu Z., Wang B., Zhao S., Sun C., Wu Y., Li Y., Ying H., Liu H. (2021). Saringosterol from Sargassum Fusiforme Modulates Cholesterol Metabolism and Alleviates Atherosclerosis in ApoE-Deficient Mice. Mar. Drugs.

[80] Eguchi K., Fujiwara Y., Hayashida A., Horlad H., Kato H., Rotinsulu H., Losung F., Mangindaan R.E.P., De Voogd N.J., Takeya M. (2013). Manzamine A, a Marine-Derived Alkaloid, Inhibits Accumulation of Cholesterol Ester in Macrophages and Suppresses Hyperlipidemia and Atherosclerosis In Vivo. Bioorganic Med. Chem..

[81] Yang Y., Seo J.M., Nguyen A., Pham T.X., Park H.J., Park Y., Kim B., Bruno R.S., Lee J. (2011). Astaxanthin-Rich Extract from the Green Alga Haematococcus Pluvialis Lowers Plasma Lipid Concentrations and Enhances Antioxidant Defense in Apolipoprotein E Knockout Mice. J. Nutr..

[82] Chen M.F., Hsu H.C., Liau C.S., Lee Y.T. (1999). The Role of Vitamin E on the Anti-Atherosclerotic Effect of Fish Oil in Diet-Induced Hypercholesterolemic Rabbits. Prostaglandins Other Lipid Mediat..

[83] Ampofo E., Später T., Müller I., Eichler H., Menger M.D., Laschke M.W. (2015). The Marine-Derived Kinase Inhibitor Fascaplysin Exerts Anti-Thrombotic Activity. Mar. Drugs.

[84] Pan N., Li Z.C., Li Z.H., Chen S.H., Jiang M.H., Yang H.Y., Liu Y.S., Hu R., Zeng Y.W., Dai L.H. (2022). Antiplatelet and Antithrombotic Effects of Isaridin E Isolated from the Marine-Derived Fungus via Downregulating the PI3K/Akt Signaling Pathway. Mar. Drugs.

[85] Bakir E.M., Younis N.S., Mohamed M.E., El Semary N.A. (2018). Cyanobacteria as Nanogold Factories: Chemical and Anti-Myocardial Infarction Properties of Gold Nanoparticles Synthesized by Lyngbya Majuscula. Mar. Drugs.

[86] Xiao Y.F., Sigg D.C., Ujhelyi M.R., Wilhelm J.J., Richardson E.S., Iaizzo P.A. (2008). Pericardial Delivery of Omega-3 Fatty Acid: A Novel Approach to Reducing Myocardial Infarct Sizes and Arrhythmias. Am. J. Physiol. Heart Circ. Physiol..

[87] Desnoyers M., Gilbert K., Rousseau G. (2018). Cardioprotective Effects of Omega-3 Polyunsaturated Fatty Acids: Dichotomy between Experimental and Clinical Studies. Mar. Drugs.

[88] Pan N., Lu L.Y., Li M., Wang G.H., Sun F.Y., Sun H.S., Wen X.J., Cheng J.D., Chen J.W., Pang J.Y. (2017). Xyloketal B Alleviates Cerebral Infarction and Neurologic Deficits in a Mouse Stroke Model by Suppressing the ROS/TLR4/NF-Î° B Inflammatory Signaling Pathway. Acta Pharmacol. Sin..

[89] Sergeevichev D., Fomenko V., Strelnikov A., Dokuchaeva A., Vasilieva M., Chepeleva E., Rusakova Y., Artemenko S., Romanov A., Salakhutdinov N. (2020). Botulinum Toxin-Chitosan Nanoparticles Prevent Arrhythmia in Experimental Rat Models. Mar. Drugs.

[90] Kang J.X., Leaf A. (2000). Prevention of Fatal Cardiac Arrhythmias by Polyunsaturated Fatty Acids. Am. J. Clin. Nutr..

[91] Chiang Y.F., Tsai C.H., Chen H.Y., Wang K.L., Chang H.Y., Huang Y.J., Hong Y.H., Ali M., Shieh T.M., Huang T.C. (2021). Protective Effects of Fucoxanthin on Hydrogen Peroxide-Induced Calcification of Heart Valve Interstitial Cells. Mar. Drugs.

[92] El-Baz F.K., Hussein R.A., Saleh D.O., Jaleel G.A.R.A. (2019). Zeaxanthin Isolated from Dunaliella Salina Microalgae Ameliorates Age Associated Cardiac Dysfunction in Rats through Stimulation of Retinoid Receptors. Mar. Drugs.

[93] Soehnlein O., Libby P. (2021). Targeting Inflammation in Atherosclerosis–from Experimental Insights to the Clinic. Nat. Rev. Drug Discov..

[94] Patil N.P., Le V., Sligar A.D., Mei L., Chavarria D., Yang E.Y., Baker A.B. (2018). Algal Polysaccharides as Therapeutic Agents for Atherosclerosis. Front. Cardiovasc. Med..

[95] Chen Z., Liu J., Fu Z., Ye C., Zhang R., Song Y., Zhang Y., Li H., Ying H., Liu H. (2014). 24(S)-Saringosterol from Edible Marine Seaweed Sargassum Fusiforme Is a Novel Selective LXRβ Agonist. J. Agric. Food Chem..

[96] Munekata P.E.S., Pateiro M., Conte-Junior C.A., Domínguez R., Nawaz A., Walayat N., Fierro E.M., Lorenzo J.M. (2021). Marine Alkaloids: Compounds with in Vivo Activity and Chemical Synthesis. Mar. Drugs.

[97] Fassett R.G., Coombes J.S. (2011). Astaxanthin: A Potential Therapeutic Agent in Cardiovascular Disease. Mar. Drugs.

[98] D’Alessandro E., Becker C., Bergmeier W., Bode C., Bourne J.H., Brown H., Buller H.R., Ten Cate-Hoek A.J., Ten Cate V., Van Cauteren Y.J.M. (2020). Thrombo-Inflammation in Cardiovascular Disease: An Expert Consensus Document from the Third Maastricht Consensus Conference on Thrombosis. Thromb Haemost.

[99] Lordan R., Tsoupras A., Zabetakis I. (2021). Platelet Activation and Prothrombotic Mediators at the Nexus of Inflammation and Atherosclerosis: Potential Role of Antiplatelet Agents. Blood Rev..

[100] Palur Ramakrishnan A.V.K., Varghese T.P., Vanapalli S., Nair N.K., Mingate M.D. (2017). Platelet Activating Factor: A Potential Biomarker in Acute Coronary Syndrome?. Cardiovasc. Ther..

[101] Soni R., Muller L., Furet P., Schoepfer J., Stephan C., Zumstein-Mecker S., Fretz H., Chaudhuri B. (2000). Inhibition of Cyclin-Dependent Kinase 4 (Cdk4) by Fascaplysin, a Marine Natural Product. Biochem. Biophys. Res. Commun..

[102] Jiang M., Wu Z., Wu Q., Yin H., Guo H., Yuan S., Liu Z., Chen S., Liu L. (2021). Amphichoterpenoids A–C, Unprecedented Picoline-Derived Meroterpenoids from the Ascidian-Derived Fungus Amphichorda Felina SYSU-MS7908. Chin. Chem. Lett..

[103] Ren R., Azuma Y., Ojima T., Hashimoto T., Mizuno M., Nishitani Y., Yoshida M., Azuma T., Kanazawa K. (2013). Modulation of Platelet Aggregation-Related Eicosanoid Production by Dietary F-Fucoidan from Brown Alga Laminaria Japonica in Human Subjects. Br. J. Nutr..

[104] Sakamoto A., Saotome M., Iguchi K., Maekawa Y. (2019). Marine-Derived Omega-3 Polyunsaturated Fatty Acids and Heart Failure: Current Understanding for Basic to Clinical Relevance. Int. J. Mol. Sci..

[105] Wu Y., Chang T., Chen W., Wang X., Li J., Chen Y., Yu Y., Shen Z., Yu Q., Zhang Y. (2021). Release of VEGF and BMP9 from Injectable Alginate Based Composite Hydrogel for Treatment of Myocardial Infarction. Bioact. Mater..

[106] Wu T., Liu W. (2022). Functional Hydrogels for the Treatment of Myocardial Infarction. NPG Asia Mater..

[107] Fan C., Joshi J., Li F., Xu B., Khan M., Yang J., Zhu W. (2020). Nanoparticle-Mediated Drug Delivery for Treatment of Ischemic Heart Disease. Front. Bioeng. Biotechnol..

[108] Ogita H., Node K., Asanuma H., Sanada S., Takashima S., Minamino T., Soma M., Kim J., Hori M., Kitakaze M. (2003). Eicosapentaenoic Acid Reduces Myocardial Injury Induced by Ischemia and Reperfusion in Rabbit Hearts. J. Cardiovasc. Pharmacol..

[109] Severino P., D’Amato A., Prosperi S., Magnocavallo M., Mariani M.V., Netti L., Birtolo L.I., De Orchi P., Chimenti C., Maestrini V. (2021). Potential Role of ENOS Genetic Variants in Ischemic Heart Disease Susceptibility and Clinical Presentation. J. Cardiovasc. Dev. Dis..

[110] Khan M.A., Hashim M.J., Mustafa H., Baniyas M.Y., Al Suwaidi S.K.B.M., AlKatheeri R., Alblooshi F.M.K., Almatrooshi M.E.A.H., Alzaabi M.E.H., Al Darmaki R.S. (2020). Global Epidemiology of Ischemic Heart Disease: Results from the Global Burden of Disease Study. Cureus.

[111] Papier K., Knuppel A., Syam N., Jebb S.A., Key T.J. (2023). Meat Consumption and Risk of Ischemic Heart Disease: A Systematic Review and Meta-Analysis. Crit. Rev. Food Sci. Nutr..

[112] Park J.H., Lee N.K., Lim H.J., Mazumder S., Rethineswaran V.K., Kim Y.J., Jang W.B., Ji S.T., Kang S., Kim D.Y. (2019). Therapeutic Cell Protective Role of Histochrome under Oxidative Stress in Human Cardiac Progenitor Cells. Mar. Drugs.

[113] Dwivedi R., Pomin V.H. (2020). Marine Antithrombotics. Mar. Drugs.

[114] Zhong R., Wan X., Wang D., Zhao C., Liu D., Gao L., Wang M., Wu C.J., Nabavid S.M., Daglia M. (2020). Polysaccharides from Marine Enteromorpha: Structure and Function. Trends Food Sci. Technol..

[115] Akil L., Anwar Ahmad H. (2011). Relationships between Obesity and Cardiovascular Diseases in Four Southern States and Colorado. J. Health Care Poor Underserved.

[116] Bhatia S., Makkar R., Behl T., Sehgal A., Singh S., Rachamalla M., Mani V., Iqbal M.S., Bungau S.G. (2022). Biotechnological Innovations from Ocean: Transpiring Role of Marine Drugs in Management of Chronic Disorders. Molecules.

[117] Zhao J., Cao Q., Xing M., Xiao H., Cheng Z., Song S., Ji A. (2020). Advances in the Study of Marine Products with Lipid-Lowering Properties. Mar. Drugs.

[118] Riccioni G., D’Orazio N., Franceschelli S., Speranza L. (2011). Marine Carotenoids and Cardiovascular Risk Markers. Mar. Drugs.

[119] Kishimoto Y., Yoshida H., Kondo K. (2016). Potential Anti-Atherosclerotic Properties of Astaxanthin. Mar. Drugs.

[120] Chen W.L., Qian Y., Meng W.F., Pang J.Y., Lin Y.C., Guan Y.Y., Chen S.P., Liu J., Pei Z., Wang G.L. (2009). A Novel Marine Compound Xyloketal B Protects against Oxidized LDL-Induced Cell Injury in Vitro. Biochem. Pharmacol..

[121] Blackwell D.J., Schmeckpeper J., Knollmann B.C. (2022). Animal Models to Study Cardiac Arrhythmias. Circ. Res..

[122] Do H.K., Kogure K., Imada C., Noguchi T., Ohwada K., Simidu U. (1991). Tetrodotoxin Production of Actinomycetes Isolated from Marine Sediment. J. Appl. Bacteriol..

[123] Hong B., He J., Le Q., Bai K., Chen Y., Huang W. (2019). Combination Formulation of Tetrodotoxin and Lidocaine as a Potential Therapy for Severe Arrhythmias. Mar. Drugs.

[124] Mackieh R., Abou-Nader R., Wehbe R., Mattei C., Legros C., Fajloun Z., Sabatier J.M. (2021). Voltage-Gated Sodium Channels: A Prominent Target of Marine Toxins. Mar. Drugs.

[125] Saravanan P., Davidson N.C., Schmidt E.B., Calder P.C. (2010). Cardiovascular Effects of Marine Omega-3 Fatty Acids. Lancet.

[126] Bali J., Thakur R. (2005). Poison as Cure: A Clinical Review of Botulinum Toxin as an Invaluable Drug. J. Venom. Anim. Toxins Incl. Trop. Dis..

[127] Ertl G., Gaudran P., Neubauer S., Bauer B., Horn M., Hu K., Tian R. (1993). Cardiac Dysfunction and Development of Heart Failure. Eur. Heart J..

[128] Rhee E.J., Plutzky J. (2012). Retinoid Metabolism and Diabetes Mellitus. Diabetes Metab. J..

[129] Tang X.H., Gudas L.J. (2011). Retinoids, Retinoic Acid Receptors, and Cancer. Annu. Rev. Pathol. Mech. Dis..

[130] Shao M., Lu L., Wang Q., Ma L., Tian X., Li C., Li C., Guo D., Wang Q., Wang W. (2021). The Multi-Faceted Role of Retinoid X Receptor in Cardiovascular Diseases. Biomed. Pharmacother..

[131] Zhu S., Guleria R.S., Thomas C.M., Roth A., Gerilechaogetu F., Kumar R., Dostal D.E., Baker K.M., Pan J. (2016). Loss of Myocardial Retinoic Acid Receptor α Induces Diastolic Dysfunction by Promoting Intracellular Oxidative Stress and Calcium Mishandling in Adult Mice. J. Mol. Cell. Cardiol..

[132] Guleria R.S., Singh A.B., Nizamutdinova I.T., Souslova T., Mohammad A.A., Kendall J.A., Baker K.M., Pan J. (2013). Activation of Retinoid Receptor-Mediated Signaling Ameliorates Diabetes-Induced Cardiac Dysfunction in Zucker Diabetic Rats. J. Mol. Cell. Cardiol..

[133] Méresse S., Fodil M., Fleury F., Chénais B. (2020). Fucoxanthin, a Marine-Derived Carotenoid from Brown Seaweeds and Microalgae: A Promising Bioactive Compound for Cancer Therapy. Int. J. Mol. Sci..

[134] Liu X., Shibata T., Hisaka S., Osawa T. (2009). Astaxanthin Inhibits Reactive Oxygen Species-Mediated Cellular Toxicity in Dopaminergic SH-SY5Y Cells via Mitochondria-Targeted Protective Mechanism. Brain Res..

[135] Fakhri S., Aneva I.Y., Farzaei M.H., Sobarzo-Sánchez E. (2019). The Neuroprotective Effects of Astaxanthin: Therapeutic Targets and Clinical Perspective. Molecules.

[136] Ikeda Y., Tsuji S., Satoh A., Ishikura M., Shirasawa T., Shimizu T. (2008). Protective Effects of Astaxanthin on 6-Hydroxydopamine-Induced Apoptosis in Human Neuroblastoma SH-SY5Y Cells. J. Neurochem..

[137] Suleria H.A.R., Gobe G., Masci P., Osborne S.A. (2016). Marine Bioactive Compounds and Health Promoting Perspectives; Innovation Pathways for Drug Discovery. Trends Food Sci. Technol..

[138] Cardoso S.M., Pereira O.R., Seca A.M.L., Pinto D.C.G.A., Silva A.M.S. (2015). Seaweeds as Preventive Agents for Cardiovascular Diseases: From Nutrients to Functional Foods. Mar. Drugs.

[139] Spoială A., Ilie C.I., Ficai D., Ficai A., Andronescu E. (2022). From Biomedical Applications of Alginate towards CVD Implications Linked to COVID-19. Pharmaceuticals.

[140] Xu Z., Lam M.T. (2018). Alginate Application for Heart and Cardiovascular Diseases. Springer Ser. Biomater. Sci. Eng..

[141] Sokolova E.V., Bogdanovich L.N., Ivanova T.B., Byankina A.O., Kryzhanovskiy S.P., Yermak I.M. (2014). Effect of Carrageenan Food Supplement on Patients with Cardiovascular Disease Results in Normalization of Lipid Profile and Moderate Modulation of Immunity System Markers. PharmaNutrition.

[142] Gioele C., Marilena S., Valbona A., Nunziacarla S., Andrea S., Antonio M. (2017). Gracilaria Gracilis, Source of Agar: A Short Review. Curr. Org. Chem..

[143] Zhang B., Zhang X. (2013). Separation and Nanoencapsulation of Antitumor Polypeptide from Spirulina Platensis. Biotechnol. Prog..

[144] Cian R.E., Martínez-Augustin O., Drago S.R. (2012). Bioactive Properties of Peptides Obtained by Enzymatic Hydrolysis from Protein Byproducts of Porphyra Columbina. Food Res. Int..

[145] Furuta T., Miyabe Y., Yasui H., Kinoshita Y., Kishimura H. (2016). Angiotensin I Converting Enzyme Inhibitory Peptides Derived from Phycobiliproteins of Dulse Palmaria Palmata. Mar. Drugs.

[146] Harnedy P.A., O’Keeffe M.B., Fitzgerald R.J. (2015). Purification and Identification of Dipeptidyl Peptidase (DPP) IV Inhibitory Peptides from the Macroalga Palmaria Palmata. Food Chem..

[147] You H.N., Lee H.A., Park M.H., Lee J.H., Han J.S. (2015). Phlorofucofuroeckol A Isolated from Ecklonia Cava Alleviates Postprandial Hyperglycemia in Diabetic Mice. Eur. J. Pharmacol..

[148] Lozano-Muñoz I., Muñoz S., Díaz N.F., Medina A., Bazaes J., Riquelme C. (2020). Nutritional Enhancement of Farmed Salmon Meat via Non-GMO Nannochloropsis Gaditana: Eicosapentaenoic Acid (EPA, 20:5 n-3), Docosapentaenoic Acid (DPA, 22:5 n-3) and Vitamin D3 for Human Health. Molecules.

[149] Rayaroth A.C., Tomar R.S., Mishra R.K. (2017). Arachidonic Acid Synthesis in Mortierella Alpina: Origin, Evolution and Advancements. Proc. Natl. Acad. Sci. India Sect. B–Biol. Sci..

[150] Agarwal S., Chauhan K. (2019). Fucoidan: A Promising Target for Dyslipidemia-A Concise Review. Pharma Innov. J..

[151] Kordjazi M., Etemadian Y., Shabanpour B., Pourashouri P. (2019). Chemical Composition Antioxidant and Antimicrobial Activities of Fucoidan Extracted from Two Species of Brown Seaweeds (Sargassum Ilicifolium and S.Angustifolium) around Qeshm Island. Iran. J. Fish. Sci..

[152] Mattei C. (2018). Tetrodotoxin, a Candidate Drug for Nav1.1-Induced Mechanical Pain?. Mar. Drugs.

[153] González-Cano R., Tejada M.Á., Artacho-Cordón A., Nieto F.R., Entrena J.M., Wood J.N., Cendán C.M. (2017). Effects of Tetrodotoxin in Mouse Models of Visceral Pain. Mar. Drugs.

[154] Biessy L., Smith K.F., Wood S.A., Tidy A., van Ginkel R., Bowater J.R.D., Hawes I. (2021). A Microencapsulation Method for Delivering Tetrodotoxin to Bivalves to Investigate Uptake and Accumulation. Mar. Drugs.

[155] Durán-Riveroll L.M., Cembella A.D. (2017). Guanidinium Toxins and Their Interactions with Voltage-Gated Sodium Ion Channels. Mar. Drugs.

[156] Xiao A.J., Chen W., Xu B., Liu R., Turlova E., Barszczyk A., Sun C.L., Liu L., Deurloo M., Wang G.L. (2015). Marine Compound Xyloketal B Reduces Neonatal Hypoxic-Ischemic Brain Injury. Mar. Drugs.

[157] Tsoupras A., Brummell C., Kealy C., Vitkaitis K., Redfern S., Zabetakis I. (2022). Cardio-Protective Properties and Health Benefits of Fish Lipid Bioactives; The Effects of Thermal Processing. Mar. Drugs.

[158] Lordan R., Redfern S., Tsoupras A., Zabetakis I. (2020). Inflammation and Cardiovascular Disease: Are Marine Phospholipids the Answer?. Food Funct..

[159] Tsoupras A., Lordan R., Zabetakis I. (2018). Inflammation, Not Cholesterol, Is a Cause of Chronic Disease. Nutrients.

[160] Nasopoulou C., Nomikos T., Demopoulos C.A., Zabetakis I. (2007). Comparison of Antiatherogenic Properties of Lipids Obtained from Wild and Cultured Sea Bass (Dicentrarchus Labrax) and Gilthead Sea Bream (Sparus Aurata). Food Chem..

[161] Panayiotou A., Samartzis D., Nomikos T., Fragopoulou E., Karantonis H.C., Demopoulos C.A., Zabetakis I. (2000). Lipid Fractions with Aggregatory and Antiaggregatory Activity toward Platelets in Fresh and Fried Cod (Gadus Morhua): Correlation with Platelet-Activating Factor and Atherogenesis. J. Agric. Food Chem..

[162] De Leonardis A., Macciola V. (2004). A Study on the Lipid Fraction of Adriatic Sardine Filets (Sardina Pilchardus). Nahrung–Food.

[163] Conde T.A., Zabetakis I., Tsoupras A., Medina I., Costa M., Silva J., Neves B., Domingues P., Domingues M.R. (2021). Microalgal Lipid Extracts Have Potential to Modulate the Inflammatory Response: A Critical Review. Int. J. Mol. Sci..

[164] Koukouraki P., Tsoupras A., Sotiroudis G., Demopoulos C.A., Sotiroudis T.G. (2020). Antithrombotic Properties of Spirulina Extracts against Platelet-Activating Factor and Thrombin. Food Biosci..

[165] Nasopoulou C., Tsoupras A.B., Karantonis H.C., Demopoulos C.A., Zabetakis I. (2011). Fish Polar Lipids Retard Atherosclerosis in Rabbits by Down-Regulating PAF Biosynthesis and up-Regulating PAF Catabolism. Lipids Health Dis..

[166] Burri L., Hoem N., Banni S., Berge K. (2012). Marine Omega-3 Phospholipids: Metabolism and Biological Activities. Int. J. Mol. Sci..

[167] Fosshaug L.E., Berge R.K., Beitnes J.O., Berge K., Vik H., Aukrust P., Gullestad L., Vinge L.E., Oie E. (2011). Krill Oil Attenuates Left Ventricular Dilatation after Myocardial Infarction in Rats. Lipids Health Dis..

[168] Granger D.N., Rodrigues S.F., Yildirim A., Senchenkova E.Y. (2010). Microvascular Responses to Cardiovascular Risk Factors. Microcirculation.

[169] Verouti S.N., Tsoupras A.B., Alevizopoulou F., Demopoulos C.A., Iatrou C. (2013). Paricalcitol Effects on Activities and Metabolism of Platelet Activating Factor and on Inflammatory Cytokines in Hemodialysis Patients. Int. J. Artif. Organs.

[170] de la Guía-Galipienso F., Martínez-Ferran M., Vallecillo N., Lavie C.J., Sanchis-Gomar F., Pareja-Galeano H. (2021). Vitamin D and Cardiovascular Health. Clin. Nutr..

[171] Nestel P.J. (2000). Fish Oil and Cardiovascular Disease: Lipids and Arterial Function. Am. J. Clin. Nutr..

[172] Kander M.C., Cui Y., Liu Z. (2017). Gender Difference in Oxidative Stress: A New Look at the Mechanisms for Cardiovascular Diseases. J. Cell. Mol. Med..

[173] Moris D., Spartalis M., Spartalis E., Karachaliou G.S., Karaolanis G.I., Tsourouflis G., Tsilimigras D.I., Tzatzaki E., Theocharis S. (2017). The Role of Reactive Oxygen Species in the Pathophysiology of Cardiovascular Diseases and the Clinical Significance of Myocardial Redox. Ann. Transl. Med..

[174] Guerby P., Tasta O., Swiader A., Pont F., Bujold E., Parant O., Vayssiere C., Salvayre R., Negre-Salvayre A. (2021). Role of Oxidative Stress in the Dysfunction of the Placental Endothelial Nitric Oxide Synthase in Preeclampsia. Redox Biol..

[175] Augusti P.R., Conterato G.M.M., Somacal S., Sobieski R., Quatrin A., Maurer L., Rocha M.P., Denardin I.T., Emanuelli T. (2009). Astaxanthin Reduces Oxidative Stress, but Not Aortic Damage in Atherosclerotic Rabbits. J. Cardiovasc. Pharmacol. Ther..

[176] Cao Q., Zhao J., Xing M., Xiao H., Zhang Q., Liang H., Ji A., Song S. (2020). Current Research Landscape of Marine-Derived Anti-Atherosclerotic Substances. Mar. Drugs.

[177] Akram W., Tagde P., Ahmed S., Arora S., Emran T.B., Babalghith A.O., Sweilam S.H., Simal-Gandara J. (2023). Guaiazulene and Related Compounds: A Review of Current Perspective on Biomedical Applications. Life Sci..

[178] Cong L., Ren Y., Hou T., Han X., Dong Y., Wang Y., Zhang Q., Liu R., Xu S., Wang L. (2020). Use of Cardiovascular Drugs for Primary and Secondary Prevention of Cardiovascular Disease Among Rural-Dwelling Older Chinese Adults. Front. Pharmacol..

[179] Papon N., Copp B.R., Courdavault V. (2022). Marine Drugs: Biology, Pipelines, Current and Future Prospects for Production. Biotechnol. Adv..

[180] Rihan M., Sharma S.S. (2022). Role of Pyruvate Kinase M2 (PKM2) in Cardiovascular Diseases. J. Cardiovasc. Transl. Res..

